# Micro-Interface Slip Damping in a Compressed Coir Vibration Isolator

**DOI:** 10.3390/ma18194521

**Published:** 2025-09-29

**Authors:** Jem A. Rongong, Jin-Song Pei, Joseph P. Wright, Gerald A. Miller

**Affiliations:** 1 School of Mechanical, Aerospace and Civil Engineering, University of Sheffield, Sheffield S1 3JD, UK; j.a.rongong@sheffield.ac.uk; 2 School of Civil Engineering and Environmental Science, University of Oklahoma, Norman, OK 73019, USA; gamiller@ou.edu; 3 Weidlinger Applied Science, New York, NY 10005, USA; jwright.wai.com@verizon.net

**Keywords:** micro-interface slip damping, displacement ratcheting, cyclic behavior, time-varying, hysteresis, mem-models, absement, generalized momentum, extended Masing model

## Abstract

The micro-interface slip damping mechanism is insensitive to temperature, making it suitable for applications where the operating environment makes viscoelastic polymers ineffective. Damping material systems that rely on micro-interface slip typically embody randomly disposed interlocking units leading to complex material behaviors. This work studies a compressed coir vibration isolator that provides a lightweight, low cost and environmentally friendly alternative to common polymer devices. Under cyclic loading, it displays highly nonlinear hysteresis and a gradual change in properties based on the load history. The nonlinear hysteresis is captured with a Masing model, which has been shown to provide an adequate phenomenological representation of systems with large numbers of miniature stick-slip contacts. This study further explores a new way to enrich the Masing model by encoding time evolution using restoring force or displacement time integral, directly adopted from mem-models, a new family of models transferred from electrical engineering. In addition to using the data from the coir isolator, two additional datasets from clayey soil, another application of micro-interface slip damping, are used to validate the modeling approach.

## 1. Introduction

### 1.1. Background

Damping materials that are used for suppressing vibrations in machinery are commonly made from polymers and rely on their viscoelasticity. They are unsuitable for some applications because polymer viscoelasticity is effective over relatively narrow ranges of temperature and frequency [1]. One alternative is micro-interface slip damping which can be observed in thermally sprayed ceramic coatings [2], tangled metal wire devices [3], multi-strand cables [4], and granular systems [5]. Here, vibration energy is dissipated via sliding friction between many interfaces that have length scales ranging from micrometers to a few millimeters. This study considers a newcomer into this family, compressed coir.

Coir is a fibrous material found between the outer skin of a coconut and the hard inner shell (e.g., [6]). As a byproduct of the food industry, coir has traditionally found use in upholstery, ropes, matting, and compost. Individual coir fibers are light, tough, and strong, and there is growing interest in their use as the reinforcement in sustainable composites. The application considered here is different in that it involves a vibration isolator constructed from dry coir fibers without any binding matrix. It is in some ways a sustainable analogy to the tangled metal wire devices that have been the focus of significant research activity over the last decade [7].

### 1.2. Overview of This Study

A lightweight, low-cost, environmentally friendly vibration isolator is constructed from coir (dry coconut fiber) that has been pressed in a mold to form a porous solid. While effective over a broad temperature range (consistent performance seen when tested over the range 0–70 °C), properties vary with load intensity and history, making traditional modeling approaches cumbersome. This study uses load-deflection information collected from a long-duration cyclic compression test on a coir isolator. While it is known that there is considerable sample-to-sample variability, the general trends in the behavior remain the same, allowing the suitability of the model to be evaluated with this dataset.

The coir dataset does not have the time histories completely measured, which is typical in engineering reality for lengthy cyclic tests. To overcome this significant technical challenge, the required time histories are approximated by linear interpolation. A high sampling rate used for the measured segmented time histories of a cycle test makes this approximation feasible.

To provide additional support in demonstrating and justifying the proposed modeling approach, another micro-interface damping application from clayey soil is used. For the supplementary datasets, complete time histories are measured; thus, no numerical interpolation is needed [8,9].

The modeling approach seeks to capture the history dependency of the shape and location of the hysteresis loops over the duration of the test. The coir isolator test reveals other subtle features that the proposed model cannot handle, motivating future modeling work.

Two levels of modeling are involved in this inaugural modeling work: The coir isolator dataset will use restoring force–displacement, while the soil dataset will use stress–strain relations for constitutive modeling. Engineering stress and engineering strain are used so that the conversion between these two levels is straightforward.

### 1.3. Technical Challenge and Solution Strategy

The modeling challenge associated with micro-interface slip damping involves highly nonlinear load-deformation behavior, and gradual, usage-induced changes to their structure. A recent review paper of damping models for structures with mechanical joints [10] is relevant to this study. “Microslip typically occurs near the edges of the joints and is often associated with wear between the components. Moreover, the presence of the interface leads to an observed loss of stiffness as the response amplitude increases and the load across the frictional interfaces increases.” Although reference [10] refers to classical microslip in joints, where the material is considered nominally homogeneous rather than fragmented, it is relevant to this work because it reviews both rate-independent models and rate-dependent models to capture damping “through friction over highly localized microscale regions near connection points, and are known to exhibit history dependent, or hysteretic behavior”. In this work we represent hysteretic friction damping using the Masing models under the rate-independent smooth hysteretic systems, which also include the Duhem models.

As inspired by structures built from rods, wires, or strands, the Masing model [11] is an insightful choice for macroscopic modeling and simplified analysis (see [12]). Energy dissipation comes from friction at the interfaces of the rods, wires, or strands, which can be individually modeled by a bilinear hysteresis model [13,14]. Random assemblies of multiple such constituents’ interfaces lead to smooth hysteresis reloading–unloading curves that could be conceptually captured in a Masing model, where references [15,16,17,18,19] are among the studies of modeling deteriorating hysteresis using generalized Masing models.

The extended Masing model follows [20,21,22]. The virgin loading curve starts with one particular simple form proposed in Equation (5.29) of [20] for the two levels of constitutive modeling as follows:

(1) $$\begin{array}{cc} r_{M} & =\left(1-e^{-\frac{Kx_{M}}{r_{u}}}\right)r_{u},\;\mathrm{at}\mathrm{restoring}\mathrm{force}\text{-}\mathrm{displacement}\mathrm{level} \end{array}$$

(2) $$\begin{array}{cc} \sigma_{M} & =\left(1-e^{-\frac{E\varepsilon_{M}}{\sigma_{u}}}\right)\sigma_{u},\;\mathrm{at}\mathrm{stress}–\mathrm{strain}\mathrm{level} \end{array}$$

where $r_{M}$ and $\sigma_{M}$ are the restoring force and stress of the Masing model, respectively, while $x_{M}$ and $\varepsilon_{M}$ are the displacement and strain of the Masing model, respectively. There are only two independent parameters, $r_{u}$ and *K*, or $\sigma_{u}$ and *E*, for yield strength and initial stiffness, respectively. There is another power parameter *n* in [20]; however, we use n=1 throughout this study for model parsimony.

The Masing Rule 1 (out of a total of three rules) as summarized in [12] reads “the equation of any hysteretic force deformation curve can be obtained by applying the original Masing rule to the virgin loading curve using the latest point of load reversal.” By applying the Masing Rule 1, the expressions for individual minor loops can be obtained by using the same two parameters $r_{u}$ and *K*, or $\sigma_{u}$ and *E*, as in the virgin loading curve. In this study, it is the minor loops that are directly obtained in a laboratory setting—not their corresponding virgin loading curves. These two parameters will be identified from minor loops.

**Figure 1:**
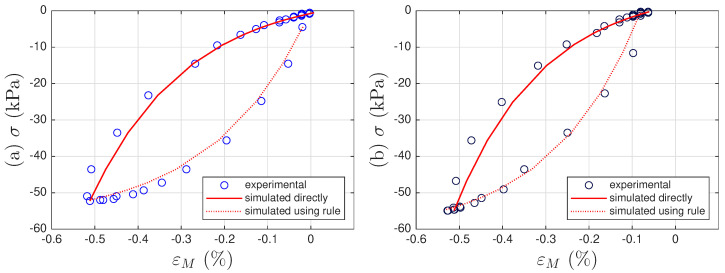
For Test 3 of the soil data: (**a**) The 11th, and (**b**) 200th intra-cycle hysteresis loops fitted using the unloading branch in Equation (40) to be presented later.

To illustrate the motivation for this study, Figure 1 presents both the experimental and modeled intra-cycle behaviors from one of the two soil datasets. The intra-cycle hysteresis loops are obtained by using the measured stress time history and the strain time history associated with intra-cycle behavior. This strain time history is obtained from a decomposition of the overall strain history into a sum of the intra- and inter-cycle time histories using the model that is presented in this work.

It can be seen from Figure 1 that the adopted extended Masing model fits the measured data adequately for each cycle. However, two different sets of parameter values for $\sigma_{u}$ and *E* are needed for these two cycles. In Figure 1a, we have $\sigma_{u}=-28.62$ kPa, and E=25.79 MPa, while in Figure 1b, we have $\sigma_{u}=-30.53$ kPa, and E=29.94 MPa. This indicates that the adopted extended Masing model needs to evolve with time due to the effect of soil compaction.

Both compressed coir fiber and soil have time-varying properties, the focus of our modeling. We will build on the extended Masing model, and inject new concepts from mem-modeling, an emerging framework for hysteresis, to develop an enriched extended Masing model, which is an autonomous model in appearance but is able to capture time-varying Masing behaviors. This modeling component is called a “mem-spring”. New concepts to be applied include the first time integral of strain and stress, called “absement” and “generalized momentum”, respectively. They enable a more efficient problem formulation for time-varying intra-cycle behaviors.

Apart from the modeling challenge for intra-cycle behaviors, there are significant and complex inter-cycle behaviors of strain-ratcheting in both coir isolator and soil data. We will utilize a “mem-dashpot”, a mem-modeling component proposed in our recent work of [23]. In short, it is a displacement-dependent damper, characterized in another efficient problem formulation for inter-cycle behaviors.

**Figure 2:**
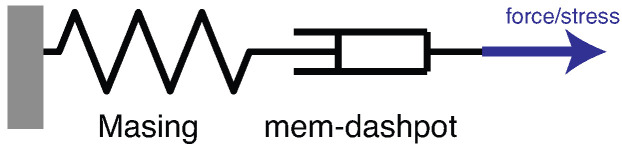
A schematic of the proposed Maxwell-like mechanism with a mem-spring and mem-dashpot connected in series, where the extended Masing model—enriched by mem-modeling to produce minor loops—is used as a mem-spring in this study.

As shown in Figure 2, the mem-spring and mem-dashpot are connected in series in this study to capture the displacement or strain ratcheting, and gradual compaction of the system, respectively. The complicated systems at hand are treated with two independent modeling components, the effects of which are decoupled based on force equilibrium and deformation compatibility and further fine-turned individually following the physical insights into intra-and inter-cycle behaviors. This model assembly follows [9,23].

Specially, the serial connectivity leads to shared restoring forces or stresses, and additivity of the displacements or strains:

(3) $$\begin{array}{cc} r & =r_{d}=r_{M},\;\mathrm{at}\mathrm{restoring}\mathrm{force}\text{-}\mathrm{displacement}\mathrm{level} \end{array}$$

(4) $$\begin{array}{cc} x & =x_{d}+x_{M},\;\mathrm{at}\mathrm{restoring}\mathrm{force}\text{-}\mathrm{displacement}\mathrm{level} \end{array}$$

(5) $$\begin{array}{cc} \sigma & =\sigma_{d}=\sigma_{M},\;\mathrm{at}\mathrm{stress}–\mathrm{strain}\mathrm{level} \end{array}$$

(6) $$\begin{array}{cc} \varepsilon & =\varepsilon_{d}+\varepsilon_{M},\;\mathrm{at}\mathrm{stress}–\mathrm{strain}\mathrm{level} \end{array}$$

where the subscript *d* stands for mem-dashpot, while the subscript *M* stands for the extended Masing model.

Identifying a model assembly like the one shown in Figure 2 demands a comprehensive testing scheme with carefully defined excitation and sampling rate [24,25]. Since the testing and modeling are not integrated in this study, the identification of the proposed model assembly has an unidentifiable difficulty [26]. We will highlight this fundamental challenge and the further assumptions that we will make in Section 4.

### 1.4. Mem-Models

“Mem-models” referring to a special form of memory, i.e., hysteresis, were introduced to engineering mechanics in [23,27,28]. Mem-models are based on a suite of new concepts for the memristor, memcapacitor, and meminductor developed for electrical engineering in [29,30], catching attention since [31], and being generalized in [32]. The publications on mem-dashpots, mem-springs, and mem-inerters followed the lead of [33,34].

The following introduction benefits from the mathematical parallelism between mem-dashpot and mem-spring; however, it is important to remember that dashpots and springs are different physically and so are mem-dashpot and mem-spring [27].

First, mem-dashpot and mem-spring can be obtained by generalizing from linear dashpot and linear spring, respectively, as follows:

(7) $$\begin{array}{cc} r_{d} & =c{\dot{x}}_{d}\to r_{d}=D\left(x_{d}\right){\dot{x}}_{d},\;\mathrm{for}\mathrm{mem}\text{-}\mathrm{dashpot}\mathrm{at}\mathrm{restoring}\mathrm{force}\text{-}\mathrm{displacement}\mathrm{level} \end{array}$$

(8) $$\begin{array}{cc} r_{s} & =kx_{s}\to r_{s}=S\left(a_{s}\right)x_{s},\;\mathrm{for}\mathrm{mem}\text{-}\mathrm{spring}\mathrm{at}\mathrm{restoring}\mathrm{force}\text{-}\mathrm{displacement}\mathrm{level} \end{array}$$

(9) $$\begin{array}{cc} \sigma_{d} & =C{\dot{\varepsilon }}_{d}\to \sigma_{d}=D\left(\varepsilon_{d}\right){\dot{\varepsilon }}_{d},\;\mathrm{for}\mathrm{mem}\text{-}\mathrm{dashpot}\mathrm{at}\mathrm{stress}–\mathrm{strain}\mathrm{level} \end{array}$$

(10) $$\begin{array}{cc} \sigma_{s} & =E\varepsilon_{s}\to \sigma_{s}=S\left(a_{s}^{\varepsilon }\right)\varepsilon_{s},\;\mathrm{for}\mathrm{mem}\text{-}\mathrm{spring}\mathrm{at}\mathrm{stress}–\mathrm{strain}\mathrm{level}, \end{array}$$

where the subscripts *d* and *s* stand for mem-dashpot and mem-spring herein, respectively. *D* and *S* are displacement- or strain-dependent damping and absement-dependent stiffness, respectively. The same notations are used for these two quantities at the two different levels only to reduce the number of notations in the presentation. *a* and $a^{\varepsilon }$ are absements defined as follows:

(11) $$\begin{array}{cc} a & =\int_{-\infty }^{t}x\left(\tau \right)d\tau ,\;\mathrm{at}\mathrm{restoring}\mathrm{force}\text{-}\mathrm{displacement}\mathrm{level}, \end{array}$$

(12) $$\begin{array}{cc} a^{\varepsilon } & =\int_{-\infty }^{t}\varepsilon \left(\tau \right)d\tau ,\;\mathrm{at}\mathrm{stress}–\mathrm{strain}\mathrm{level}, \end{array}$$

meaning that absement is a time integral of displacement or strain. Absement has not been often used in modeling. Most recently, absement has been connected quantitatively to the damage variable in continuum damage mechanics in [23]. Reference [23] further shows that a subset of the classical Preisach model can be transformed into an equivalent mem-spring model. The connection between the generalized Duhem model and mem-models is discussed there as well.

Integrating Equations (7)–(10) with respect to time leads to the following:

(13) $$\begin{array}{cc} p_{d} & =G_{d}\left(x_{d}\right),\;\mathrm{for}\mathrm{mem}\text{-}\mathrm{dashpot}\mathrm{at}\mathrm{restoring}\mathrm{force}\text{-}\mathrm{displacement}\mathrm{level} \end{array}$$

(14) $$\begin{array}{cc} p_{s} & =G_{s}\left(a_{s}\right),\;\mathrm{for}\mathrm{mem}\text{-}\mathrm{spring}\mathrm{at}\mathrm{restoring}\mathrm{force}\text{-}\mathrm{displacement}\mathrm{level} \end{array}$$

(15) $$\begin{array}{cc} p_{d}^{\sigma } & =G_{d}\left(\varepsilon_{d}\right),\;\mathrm{for}\mathrm{mem}\text{-}\mathrm{dashpot}\mathrm{at}\mathrm{stress}–\mathrm{strain}\mathrm{level} \end{array}$$

(16) $$\begin{array}{cc} p_{s}^{\sigma } & =G_{s}\left(a_{s}^{\varepsilon }\right),\;\mathrm{for}\mathrm{mem}\text{-}\mathrm{spring}\mathrm{at}\mathrm{stress}–\mathrm{strain}\mathrm{level}, \end{array}$$

where *p* and $p^{\sigma }$ are referred to as generalized momentums (“g-momentum” for shorthand notation) defined as follows:

(17) $$\begin{array}{cc} p & =\int_{-\infty }^{t}r\left(\tau \right)d\tau ,\;\mathrm{at}\mathrm{restoring}\mathrm{force}\text{-}\mathrm{displacement}\mathrm{level} \end{array}$$

(18) $$\begin{array}{cc} p^{\sigma } & =\int_{-\infty }^{t}\sigma \left(\tau \right)d\tau ,\;\mathrm{at}\mathrm{stress}–\mathrm{strain}\mathrm{level}, \end{array}$$

meaning that generalized momentum is a time integral of restoring force or stress. Generalized momentum should not be mixed with momentum.

The one-to-one mappings from $x_{d}$ to $p_{d}$, $a_{s}$ to $p_{s}$, $\varepsilon_{d}$ to $p_{d}^{\sigma }$, and $a_{s}^{\varepsilon }$ to $p_{s}^{\sigma }$ as in Equations (13)–(16) are significant to define mem-dashpot and mem-spring as we shall see more clearly using the specified data. These one-to-one mappings are nonlinear; otherwise, the mem-dashpot and mem-spring will degenerate to the linear dashpot and linear spring, respectively.

When the one-to-one mappings in Equations (13)–(16) are invertible, we have the following:

(19) $$\begin{array}{cc} x_{d} & =F_{d}\left(p_{d}\right),\;\mathrm{for}\mathrm{mem}\text{-}\mathrm{dashpot}\mathrm{at}\mathrm{restoring}\mathrm{force}\text{-}\mathrm{displacement}\mathrm{level} \end{array}$$

(20) $$\begin{array}{cc} a_{s} & =F_{s}\left(p_{s}\right),\;\mathrm{for}\mathrm{mem}\text{-}\mathrm{spring}\mathrm{at}\mathrm{restoring}\mathrm{force}\text{-}\mathrm{displacement}\mathrm{level} \end{array}$$

(21) $$\begin{array}{cc} \varepsilon_{d} & =F_{d}\left(p_{d}^{\sigma }\right),\;\mathrm{for}\mathrm{mem}\text{-}\mathrm{dashpot}\mathrm{at}\mathrm{stress}–\mathrm{strain}\mathrm{level} \end{array}$$

(22) $$\begin{array}{cc} a_{s}^{\varepsilon } & =F_{s}\left(p_{s}^{\sigma }\right),\;\mathrm{for}\mathrm{mem}\text{-}\mathrm{spring}\mathrm{at}\mathrm{stress}–\mathrm{strain}\mathrm{level}, \end{array}$$

where $F=G^{-1}$. The one-to-one mappings from $p_{d}$ to $x_{d}$, $p_{s}$ to $a_{s}$, $p_{d}^{\sigma }$ to $\varepsilon_{d}$, and $p_{s}^{\sigma }$ to $a_{s}^{\varepsilon }$ as in Equations (19)–(22) are significant to define mem-dashpot and mem-spring, respectively.

The one-to-one mappings in Equations (13)–(16) are for a displacement- or strain-controlled setting, while the one-to-one mappings in Equations (19)–(22) are for a force- or stress-controlled setting. Displacement- or strain-controlled, and force- or stress-controlled are called flow- and effort-controlled, respectively, using the jargon in bond graph theory [35,36], where the mem-models were originated.

Differentiating Equations (19)–(22) with respect to time leads to the following:

(23) $$\begin{array}{cc} {\dot{x}}_{d} & =W_{d}\left(p_{d}\right)r_{d}\;\mathrm{for}\mathrm{mem}\text{-}\mathrm{dashpot}\mathrm{at}\mathrm{restoring}\mathrm{force}\text{-}\mathrm{displacement}\mathrm{level} \end{array}$$

(24) $$\begin{array}{cc} x_{s} & =W_{s}\left(p_{s}\right)r_{s},\;\mathrm{for}\mathrm{mem}\text{-}\mathrm{spring}\mathrm{at}\mathrm{restoring}\mathrm{force}\text{-}\mathrm{displacement}\mathrm{level} \end{array}$$

(25) $$\begin{array}{cc} {\dot{\varepsilon }}_{d} & =W_{d}\left(p_{d}^{\sigma }\right)\sigma_{d}\;\mathrm{for}\mathrm{mem}\text{-}\mathrm{dashpot}\mathrm{at}\mathrm{stress}–\mathrm{strain}\mathrm{level} \end{array}$$

(26) $$\begin{array}{cc} \varepsilon_{s} & =W_{s}\left(p_{s}^{\sigma }\right)\sigma_{s}\;\mathrm{for}\mathrm{mem}\text{-}\mathrm{spring}\mathrm{at}\mathrm{stress}–\mathrm{strain}\mathrm{level}. \end{array}$$

Complementing the eight one-to-one mappings, Equations (7)–(10) are for the flow-controlled setting, while Equations (23)–(26) are for the effort-controlled setting. These eight equations manifest the “zero-crossing property” of mem-models, which is explained using two examples as follows:

- A mem-dashpot for restoring the force-displacement level can be defined in two differential forms as in Equations (7) and (23), where the former and latter are for flow- and effort-controlled settings, respectively. These two forms facilitate connecting the mem-dasphot with other modeling elements in parallel and series, respectively. These differential forms are featured with the “zero-crossing” property meaning that the input and output, $\dot{x}$ and *r*, or *r* and $\dot{x}$, become zero simultaneously. Equations (13) and (19) are the integral forms for the mem-dashpot under flow- and effort-controlled settings, respectively. These integral forms are featured with one-to-one mapping from $x_{d}$ to $p_{d}$, or $p_{d}$ to $x_{d}$. The terminologies and usefulness of the differential and integral forms can be referred to [28].
- A mem-spring for restoring the force-displacement level can be defined in two differential forms as in Equations (8) and (24) under flow- and effort-controlled settings, respectively. These differential forms are featured with the “zero-crossing” property meaning that the input and output, *x* and *r*, or *r* and *x*, become zero simultaneously. Equations (14) and (20) are the integral forms for the mem-spring under flow- and effort-controlled settings, respectively. These integral forms are featured with one-to-one mapping from $a_{s}$ to $p_{s}$, or $p_{s}$ to $a_{s}$.

Equations (7)–(26) complete the mathematical expressions for mem-dashpot and mem-spring in this study. These definitions are under mechanical memristor and mechanical memcapacitor, the simplest forms of the most comprehensive definitions for mem-dashpot and mem-spring, respectively, as elaborated in [27].

Mem-springs in this study do not have their input and output become zero simultaneously, i.e., at the origin. Rather, each of their input and output becomes a fixed value, simultaneously, i.e., these mem-springs cross at a minor loop closure point of the extended Masing model. This follows the conceptual discussion given in [23]. This will be further justified later under Section 3.

With the definitions in place, the serial connectivity illustrated in Figure 2 further leads to shared generalized momentums and additivity of the absements:

(27) $$\begin{array}{cc} p & =p_{d}=p_{M},\;\mathrm{for}\mathrm{restoring}\mathrm{force}\text{-}\mathrm{displacement}\mathrm{level} \end{array}$$

(28) $$\begin{array}{cc} a & =a_{d}+a_{M},\;\mathrm{for}\mathrm{restoring}\mathrm{force}\text{-}\mathrm{displacement}\mathrm{level} \end{array}$$

(29) $$\begin{array}{cc} p^{\sigma } & =p_{d}^{\sigma }=p_{M}^{\sigma },\;\mathrm{for}\mathrm{stress}–\mathrm{strain}\mathrm{level} \end{array}$$

(30) $$\begin{array}{cc} a^{\varepsilon } & =a_{d}^{\varepsilon }+a_{M}^{\varepsilon },\;\mathrm{for}\mathrm{stress}–\mathrm{strain}\mathrm{level}. \end{array}$$

### 1.5. Intended Contribution and Structure of This Paper

The manufacture and testing of the novel coir isolator are the first intended contribution. The coir isolator data reveals the complicated ground truth that has motivated the modeling work in this paper, and will motivate continued modeling work.

To capture time-varying Masing behaviors, the expressions for the virgin loading curve as in Equations (1) and (2) are used as a baseline before taking into account the time-varying system properties using mem-modeling concepts. The following virgin loading curves for time-varying Masing models are proposed:

At restoring force-displacement level, we have
  (31) $$r_{M}=\left(1-e^{-\frac{g_{2}\left(a_{M}\right)x_{M}}{g_{1}\left(a_{M}\right)}}\right)g_{1}\left(a_{M}\right),$$
  where $r_{u}$ and *K*, the two parameters with fixed values in Equation (1) are replaced with two functions of absement. The first function $g_{1}\left(a_{M}\right)$ is about how the yield strength is affected by absement, while the second function $g_{2}\left(a_{M}\right)$ is about how the initial stiffness is affected by absement.
At the stress–strain level, we have
  (32) $$\sigma_{M}=\left(1-e^{-\frac{f_{2}\left(p_{M}^{\sigma }\right)\varepsilon_{M}}{f_{1}\left(p_{M}^{\sigma }\right)}}\right)f_{1}\left(p_{M}^{\sigma }\right),$$
  where $\sigma_{u}$ and *E*, the two parameters with fixed values in Equation (2) are replaced with two functions of generalized momentum. The first function $f_{1}\left(p_{M}^{\sigma }\right)$ is about how the yield strength is affected by generalized momentum, while the second function $f_{2}\left(p_{M}^{\sigma }\right)$ is about how the initial stiffness is affected by generalized momentum.

These proposed models involve enriched Masing models, which are the next intended contribution.

The applicability of the Masing Rule 1 to the proposed coir isolator will be explored in this study. Limitations with applying Masing models to soil modeling were noted as early as in [37] and later in [38]. The inapplicability of the Masing Rule 1 as realized in this study is not a surprise.

As a result, two slightly different enrichment treatments will be adopted for the coir isolator and soil data. In the end, Equation (31) works well for the coir isolator data, even though the minor loops are not as accurate due to the slightly asymmetric unloading and reloading branches. Equation (32) does not work well for the virgin loading curves for the two soil datasets. We would enrich the minor loops of the extended Masing model, using them only for the two soil datasets.

While [23] details the insight into using a mechanical memristor to capture strain ratcheting in fatigue testing, joining [9], this paper numerically validates this modeling approach especially when the coir dataset only includes a small collection of typical hysteresis loops from a prolonged reload-unloading time history. This is the last intended contribution of this study, especially to motivate further studies.

Section 2 offers the design and testing details of the coir isolator. The soil specimens are also briefly introduced. Section 3 details the proposed enriched Masing model, especially those concerning the minor loops. Section 4 presents the modeling challenges, solution strategies and results of the coir isolator. Section 5 analyzes the supplementary soil specimens to further explain the proposed method. While Section 6 provides discussions, Section 7 offers concluding remarks. Appendix A supplements figures with a second soil specimen, as the main text contains the figures of just one soil specimen.

Throughout this study, numerical integration with respect to time is carried out by using the trapezoidal rule to obtain the integral counterpart for a specified input or output. Numerical differentiation with respect to time is carried out by using the central difference method. The MATLAB [39] code adopted here is central_diff.m [40] to ensure forward and backward differences at the left and right ends, respectively, and with the same second-order of accuracy as the central difference for the mid-portion.

## 2. Manufacturing and Testing

### 2.1. Coir Isolators

**Figure 3:**
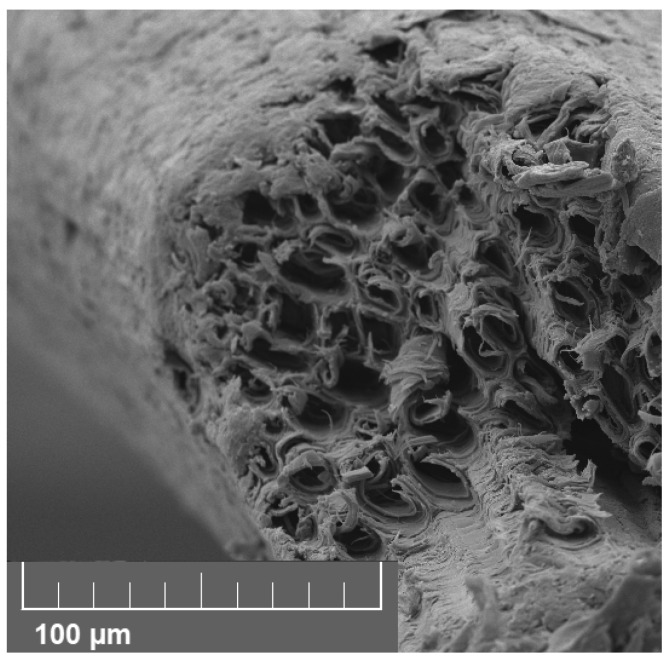
Microscope image of the broken end of a coir fiber showing the tube-like channels running along its length and the rough surface of the fiber.

The coir fibers used in this work are typically more than 25 mm in length and approximately 200 micrometers in diameter. Fiber mechanical properties are set by the materials involved, primarily cellulose and lignin, and the cell structure. Figure 3 shows that a fiber resembles a bundle of tubes that have been fused together to form a relatively smooth rod with internal pores aligned with its length. The resulting fibers typically have the following properties [41]: Young’s modulus, 4–5 GPa; strain at failure 20–40%; and density 900 kg/m^3^. As glass transition for both cellulose and lignin occurs above 100 °C, the fiber modulus does not change dramatically below this temperature.

**Figure 4:**
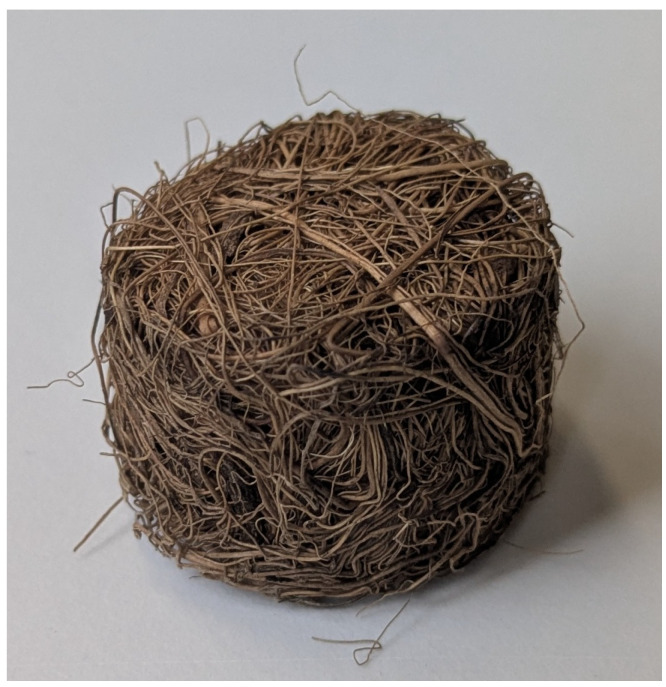
Compressed coir fiber specimen.

**Figure 5:**
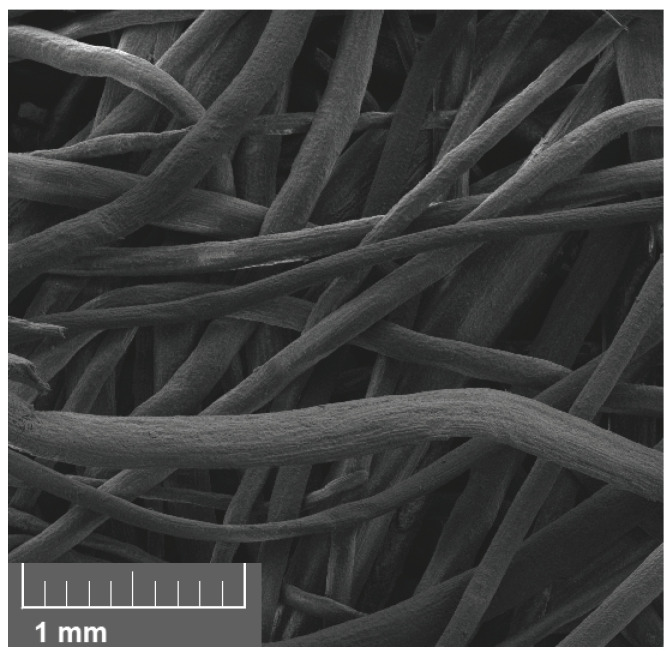
Close-up view of compressed coir fiber specimen showing typical packing and variation in fiber diameter.

The compressed coir isolators studied in this work were constructed from dry, upholstery-grade fibers. These were soaked in water for one hour and then compressed in a vented mold at 125 °C for 90 min. They were subsequently allowed to cool in the mold and then left to dry out of the mold at room temperature for 24 h. Then 2 g of fiber was compressed in a cylindrical cavity 25.4 mm in diameter and 15 mm in length, resulting in a dry fibrous solid with a relative density of approximately 0.2. A typical specimen can be seen in Figure 4, whereas the fiber packing visible on the surface can be seen more clearly in Figure 5.

For a vibration isolator application, the dynamic stiffness is of primary interest. Testing was conducted in compression, avoiding tensile loads. The stiffness was found to be insensitive to frequency over the range 1 to 100 Hz, so subsequent testing was conducted at 2.5 Hz. This initial testing suggested that the effective Young’s modulus was in the range 0.5 to 4 MPa with a loss factor between 0.1 and 0.2. The exact values depended somewhat on the particular specimen considered (due to variations in fiber size and location) and the magnitude of the loading applied. Additionally, changes in some properties were noted as testing progressed.

The response in the properties to loading intensity and history resemble, in some ways, those for tangled metal wire materials. For these, the dominant energy dissipation mechanism is sliding friction between the contacting wires [3]. In the same way, the dissipation in the compressed coir fiber is likely to be dominated by sliding at the contact fibers, although hysteretic loss within the fiber itself may provide a secondary loss mechanism. To date, there is no consensus on a compact way to model the behavior observed. The work in this paper demonstrates one way in which this can be performed.

**Figure 6:**
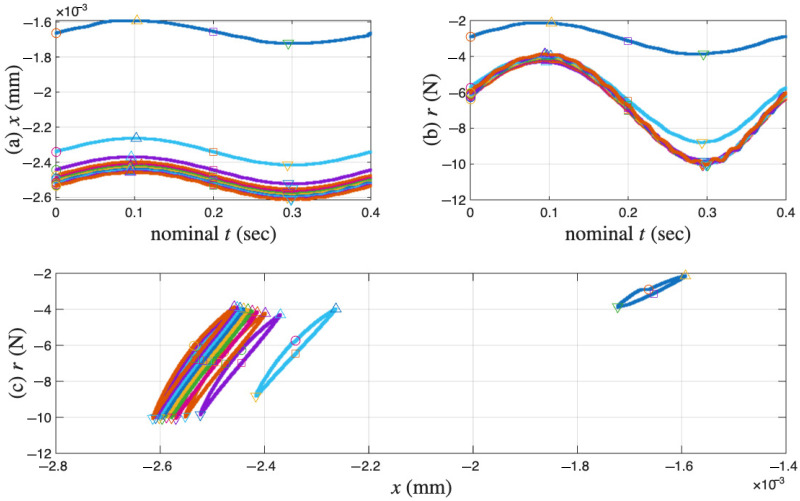
For the coir isolator: The raw data with four particular points marked in each measured cycle, which are the beginning point, maximum displacement point, the mid-point, and the minimum displacement point. The measured 11 cycles are 1, 102, 204, 300, 402, 501, 600, 702, 801, 900 and 1000. (**a**) Segmented *x* time histories, (**b**) segmented *r* time histories, and (**c**) the corresponding hysteresis loops.

Figure 6a,b present the measured displacement *x* and restoring force *r* time histories of the coir isolator in segments. Only 11 segments are measured. The sequential numbers for the measured cycles are 1, 102, 204, 300, 402, 501, 600, 702, 801, 900, and 1000. Each measured segment is of one complete cycle of 0.4 s so that the loading frequency is 2.5 Hz. The time reading is nominal because the time is reset to zero at the beginning of each segment. Figure 6c combines the time histories in Figure 6a,b for 11 isolated hysteresis loops in compression only. The sampling rate is 20 kHz. Each segment or cycle is of 8001 points. Figure 6 is plotted using only the data points; adjacent data points are not connected with a line.

It can be seen from Figure 6a,b that all displacement segments resemble a sine wave, while all restoring force segments do not. This is due to displacement-controlled testing. Four particular points of each cycle are marked so that the beginning and end points of the test as well as the loading path can be tracked visually. This is to facilitate studying hysteresis, i.e., path dependency. Each measured cycle progresses in the following manner:

(i) Starts the first point with a circle marker, where there is an approximately maximum positive velocity on an unloading branch.
(ii) Goes over the maximum displacement point, a velocity turning point to start a reloading branch. This point is not exactly the 2001st point but will be approximated using it later.
(iii) Passes the 4001st point, an approximately maximum negative velocity point marked with a square.
(iv) Reaches the minimum displacement point, the other velocity turning point to start a unloading branch. This point is not exactly the 6001st point but will be approximated using it later.
(v) Completes the cycle at the 8001st point, just before the circle marker for the next cycle (which is not measured).

It can be seen from Figure 6a,b that all maximum displacement points are approximately maximum restoring force points, and all minimum displacement points are approximately minimum restoring force points. This consistency indicates that the data satisfies the monotonicity of hysteresis, described as “vibro-correctness” in [42].

In Figure 6c, it can be seen that the cyclic test starts with negative displacement and restoring force readings, meaning that the coir isolator is in compression at the beginning of the data acquisition. This means that the virgin loading is not measured, which is good and bad news. It is good news because we have a chance to infer a virgin loading curve. It is bad news because there is no means to validate the inferred virgin loading curve.

In Figure 6c, for the unloading branches, the first cycle is not smooth in the last quarter, but all other cycles are smooth. For the reloading branches, the first cycle is smooth and curved, but all other cycles are smooth and nearly of a straight line. This observation is about the asymmetric unloading and reloading branches: the former are more curved, while the latter are straighter. This asymmetry has also been observed for tangled metal wire specimens [3]. Only the unloading branches will be used as the minor loops in this work.

Last but not least, when the excitation of x(t) is smooth, the corresponding hysteresis loop is not smooth over the velocity turning point. This indicates the hysteresis as being loading-rate independent [43]. The extended Masing model is rate independent; however, the proposed enriched Masing models in Equations (31) and (32) are not because of the use of time integrals. This is another topic for future improvement of the proposed model.

### 2.2. Clayey Soil Specimens

Two additional datasets come from the geotechnical engineering community to overcome the limitation in the coir isolator dataset. An earlier laboratory study [8] was conducted to investigate the behavior of a soft compacted clayey soil subject to cyclic loading, which was part of a comprehensive investigation of the Low Track Modulus (LTM) section. It was from the Facility for Accelerated Service Testing (FAST). Only Tests 2 and 3 datasets are studied here, and up to 200 cycles, following [9]. The measured strains of Tests 2 and 3 differ in an order of magnitude caused by the different initial degree of saturation among other factors [8]. Studying both Tests 2 and 3 data will thus validate the robustness of the proposed modeling approach. For the efficiency of presentation, the data and analysis associated with Test 3 are given in the main text, while those associated with Test 2 are shared in Appendix A.

**Figure 7:**
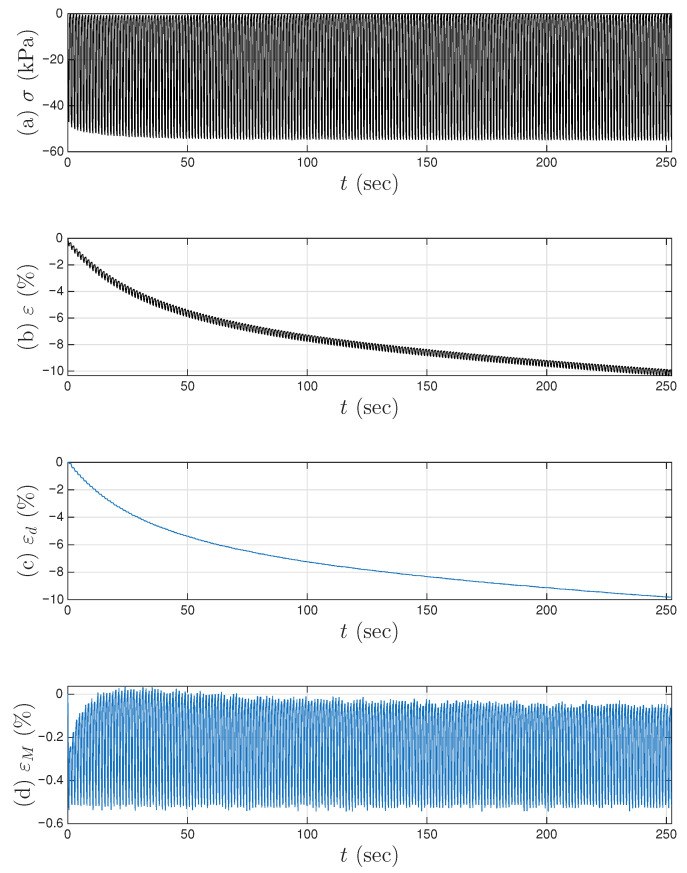
For Test 3 of the soil data: (**a**) the measured stress time history; (**b**) the measured strain time history; the decomposed strain time history for (**c**) the inter-cycle drift to be modeled by a mem-dashpot; and (**d**) the intra-cycle responses to be modeled by the enriched Masing model that is a mem-spring.

**Figure 8:**
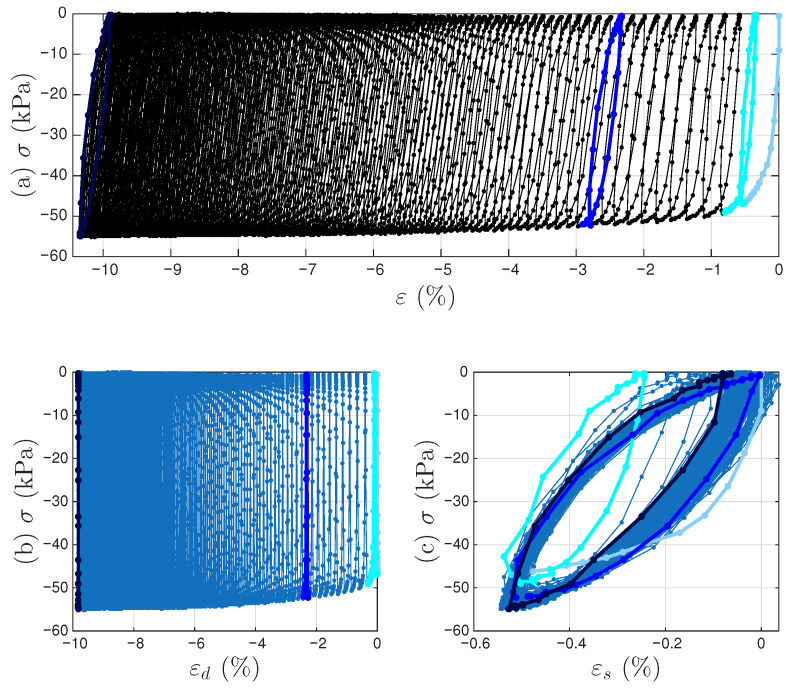
For Test 3 of the soil data: (**a**) the measured hysteresis loops of up to the 200th cycles, and the corresponding hysteresis loops to be modeled by (**b**) the mem-dashpot for the inter-cycle, and (**c**) the enriched Masing model for the intra-cycle responses resulted from decomposing the measured strain time history. The virgin loading curve, 1st, 11th, and 200th cycles are highlighted in baby blue, cyan, blue, and dark blue, respectively.

Figure 7a,b present the measured time histories for Test 3. Note that the underlying cycle-to-cycle drift in strain is significant compared with the cycle amplitude. Figure 8a shows the measured hysteresis loops in compression only for Test 3. Given the supporting role of the two soil datasets to the coir isolator dataset, Figure 8 follows Figure 6 to present compressive stress and strain in the third quadrant. Unlike the coir isolator, the measurement starts with zero strain and zero stress. If using the extended Masing model alone, we can say that the virgin loading curve is measured, and there are 200 minor loops. All these minor loops, however, do not close on the virgin loading curve. This is why the model assembly illustrated in Figure 2 will be used instead.

## 3. Proposed Models

We will show first how minor loops can be recovered from the virgin loading curve in Section 3.1. Our real intention is often to estimate the virgin loading curve from the minor loops that are measured from the real world. This is because of the two shared parameters of the extended Masing model introduced after Equations (1) and (2). We will then capture how these two parameters evolve with time by enriching the extended Masing model in Section 3.2.

### 3.1. More Review of Original Extended Masing Models

As reviewed in [12], we have the following set of equivalent expressions for minor loops of the extended Masing model whose virgin loading curve is given in Equation (1):

(33) $$\left\{\begin{array}{cc} \frac{dr_{M}}{dx_{M}} & =K\left(1-\frac{r_{M}-r^{*}}{2r_{u}}\right),\;\mathrm{unloading}\\ \frac{dr_{M}}{dx_{M}} & =K\left(1-\frac{r^{*}-r_{M}}{2r_{u}}\right),\;\mathrm{reloading} \end{array}\right.$$

(34) $$\left\{\begin{array}{cc} {\dot{r}}_{M} & =K\left(1-\frac{r_{M}-r^{*}}{2r_{u}}\right){\dot{x}}_{M},\;\mathrm{unloading}\\ {\dot{r}}_{M} & =K\left(1-\frac{r^{*}-r_{M}}{2r_{u}}\right){\dot{x}}_{M},\;\mathrm{reloading} \end{array}\right.$$

(35) $$\left\{\begin{array}{cc} r_{M} & =2r_{u}\left(1-e^{-\frac{x_{M}-x^{*}}{2x_{u}}}\right)+r^{*},\;\mathrm{unloading}\\ r_{M} & =r^{*}-2r_{u}\left(1-e^{-\frac{x^{*}-x_{M}}{2x_{u}}}\right),\;\mathrm{reloading} \end{array}\right.$$

(36) $$\left\{\begin{array}{cc} x_{M} & =-2x_{u}\ln\left(1-\frac{r_{M}-r^{*}}{2r_{u}}\right)+x^{*},\;\mathrm{unloading}\\ x_{M} & =x^{*}+2x_{u}\ln\left(1-\frac{r^{*}-r_{M}}{2r_{u}}\right),\;\mathrm{reloading} \end{array}\right.$$

where $K=\frac{r_{u}}{x_{u}}$, and $x^{*}$ and $r^{*}$ are the displacement and restoring force, respectively, at the velocity turning point where ${\dot{x}}_{M}=0$ and ${\dot{r}}_{M}=0$ simultaneously. Reloading and unloading are defined here in a physical sense, in conjunction with the fact that the responses are compressive only (so that $r_{u}<0$ and $\sigma_{u}<0$).

At the stress–strain level following the virgin loading curve given in Equation (2), we have the following equivalent expressions:

(37) $$\left\{\begin{array}{cc} \frac{d\sigma_{M}}{d\varepsilon_{M}} & =E\left(1-\frac{\sigma_{M}-\sigma^{*}}{2\sigma_{u}}\right),\;\mathrm{unloading}\\ \frac{d\sigma_{M}}{d\varepsilon_{M}} & =E\left(1-\frac{\sigma^{*}-\sigma_{M}}{2\sigma_{u}}\right),\;\mathrm{reloading} \end{array}\right.$$

(38) $$\left\{\begin{array}{cc} {\dot{\sigma }}_{M} & =E\left(1-\frac{\sigma_{M}-\sigma^{*}}{2\sigma_{u}}\right){\dot{\varepsilon }}_{M},\;\mathrm{unloading}\\ {\dot{\sigma }}_{M} & =E\left(1-\frac{\sigma^{*}-\sigma_{M}}{2\sigma_{u}}\right){\dot{\varepsilon }}_{M},\;\mathrm{reloading} \end{array}\right.$$

(39) $$\left\{\begin{array}{cc} \sigma_{M} & =2\sigma_{u}\left(1-e^{-\frac{\varepsilon_{M}-\varepsilon^{*}}{2\varepsilon_{u}}}\right)+\sigma^{*},\;\mathrm{unloading}\\ \sigma_{M} & =\sigma^{*}-2\sigma_{u}\left(1-e^{-\frac{\varepsilon^{*}-\varepsilon_{M}}{2\varepsilon_{u}}}\right),\;\mathrm{reloading} \end{array}\right.$$

(40) $$\left\{\begin{array}{cc} \varepsilon_{M} & =-2\varepsilon_{u}\ln\left(1-\frac{\sigma_{M}-\sigma^{*}}{2\sigma_{u}}\right)+\varepsilon^{*},\;\mathrm{unloading}\\ \varepsilon_{M} & =\varepsilon^{*}+2\varepsilon_{u}\ln\left(1-\frac{\sigma^{*}-\sigma_{M}}{2\sigma_{u}}\right),\;\mathrm{reloading} \end{array}\right.$$

where $E=\frac{\sigma_{u}}{\epsilon_{u}}$, and $\varepsilon^{*}$ and $\sigma^{*}$ are the displacement and restoring force, respectively, at the velocity turning point where ${\dot{\varepsilon }}_{M}=0$ and ${\dot{\sigma }}_{M}=0$ simultaneously.

**Figure 9:**
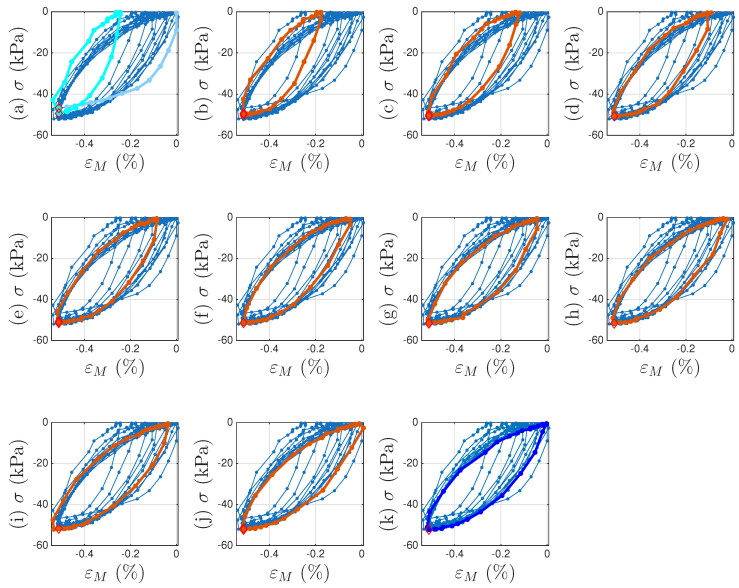
For Test 3 of the soil data: The first 11 intra-cycle hysteresis loops with one loop highlighted at a time following (**a**–**k**) to reveal the virgin loading curve and minor loop closures using Masing model concepts. The virgin loading curve, 1st and 11th intra-cycles, and other intra-cycles in between are colored in baby blue, cyan, blue, and red, respectively. The identified minor loop closure points are marked with red diamonds.

A strain decomposition method for the soil dataset following the model assembly in Figure 2 to reveal a full Masing model is presented in Section 5. The decomposed strain time histories are presented in Figure 7c,d. The strain decomposition leads to two separate hysteresis loops, one in Figure 8b for the mem-dashpot and the other in Figure 8c for the enriched Masing model. Using the first 11 intra-cycle hysteresis loops for the soil dataset as an example, Figure 9 expands Figure 8c by highlighting one loop at a time. The virgin loading curve is in Figure 9a, while the minor loop closure points marked with red diamonds are in Figure 9a–k.

The adopted extended Masing model does not fit the first intra-cycle as well as the fitting of the 11th and 200th cycle as shown in Figure 1. The numerical results of $\sigma_{u}$ and *E* and those from the minor loops are not consistent indicating that the Masing Rule 1 in [12] does not apply well. Nonetheless, Figure 9a–k shows a consistent minor loop closure point. The same minor loop closure point is thus assumed for up to the 200th intra-cycle hysteresis loops. This minor loop closure point of the Masing model could be used as the zero-crossing point of the mem-spring.

### 3.2. Proposed Enriched Extended Masing Models

Equations (33)–(40) can all be enriched using either absement or generalized momentum following Equations (31) and (32), which serve as an example for creating a time-varying Masing model. Enriching means to replace the yield strength and initial stiffness in the extended Masing model with a pair of functions of generalized momentum, or a pair of functions of absement. For the soil datasets, the former is $f_{1}\left(p_{M}^{\sigma }\right)$ and $f_{2}\left(p_{M}^{\sigma }\right)$ as in Equation (32) for a virgin loading curve. This pair of functions, $f_{1}\left(p_{M}^{\sigma }\right)$ and $f_{2}\left(p_{M}^{\sigma }\right)$, can be used to replace $\sigma_{u}$ and *E* in Equations (37)–(40) for minor loops.

**Figure 10:**
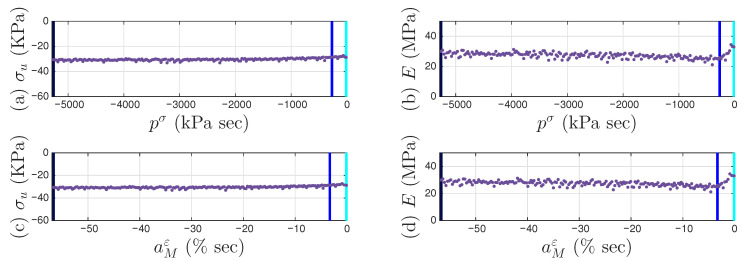
For Test 3 of the soil data: The two parameters, yield strength $\sigma_{u}$ (**a**,**c**) and initial stiffness *E* (**b**,**d**), as affected by generalized momentum $p^{\sigma }$ (**a**,**b**) and absement $a_{M}^{\varepsilon }$ (**c**,**d**) for the first 200 intra-cycles. These intra-cycles are to be modeled by a mem-spring, the enriched Masing model. The 1st, 11th, and 200th intra-cycles are colored in cyan, blue, and dark blue, respectively.

All 200 individual intra-cycles are modeled using the unloading branch in Equation (40) to obtain the values of $\sigma_{u}$ and *E* for each intra-cycle, following the illustrations in Figure 1 for the 11th and 200th intra-cycles. For each unloading branch of an intra-cycle, the value of $p^{\sigma }$ (which is equal to $p_{M}^{\sigma }$ following Equation (29)) at the beginning of the unloading branch is collected. This is the minor loop closure point, which is a salient detail. The top row of Figure 10 presents how $\sigma_{u}$ and *E* are affected by $p_{M}^{\sigma }$ for the first 200 cycles. Fitting the 200 discrete data points in each panel would lead to $f_{1}\left(p_{M}^{\sigma }\right)$ and $f_{2}\left(p_{M}^{\sigma }\right)$ in Equation (32) for an effort-controlled setting. Holding the values for $f_{1}\left(p_{M}^{\sigma }\right)$ and $f_{2}\left(p_{M}^{\sigma }\right)$ constant for the entire branch was discussed in the context of the differential algebraic equation (DAE) for a full-system dynamic simulation in [9].

For each unloading branch of an intra-cycle, the value of $a_{M}^{\varepsilon }$ at the beginning of the unloading branch is collected too. This is the minor loop closure point. The bottom row of Figure 10 presents how $\sigma_{u}$ and *E* are affected and $a_{M}^{\varepsilon }$ for the first 200 cycles. Fitting the 200 discrete data points in each panel would lead to a variation of Equation (32) for a flow-controlled setting.

The flexibility of treating each of $\sigma_{u}$ and *E* as a function of either absement or generalized momentum is enabled by the one-to-one mappings between absement and generalized momentum for a mem-spring as in Equations (16) and (22). This minor loop closure point of the Masing model will be used as the zero-crossing point of the mem-spring.

Again, enriching means to replace the yield strength and initial stiffness in the extended Masing model with a pair of functions of absement, or a pair of functions of generalized momentum. For the coir isolator datasets, the former is $g_{1}\left(a_{M}\right)$ and $g_{2}\left(a_{M}\right)$ as in Equation (31) for a virgin loading curve. This pair of functions, $g_{1}\left(a_{M}\right)$ and $g_{2}\left(a_{M}\right)$, can be used to replace $r_{u}$ and *K* in Equations (33) and (36) for minor loops. The virgin loading curve is not measured. The time histories of many minor loops are not available. Nonetheless, we will perform a data analysis for modeling and identification strategies and utilize available data measurements to construct plots as in Figure 10 but for $r_{u}$ versus $a_{M}$ and *K* versus $a_{M}$, which are $g_{1}\left(a_{M}\right)$ and $g_{2}\left(a_{M}\right)$, respectively. For $g_{1}\left(a_{M}\right)$, we will use a major assumption to be given in Section 4.1. For $g_{2}\left(a_{M}\right)$, when $r_{M}\to r^{*}$, we have $\frac{dr_{M}}{dx_{M}}\to K$ based on Equation (33).

## 4. Data Analysis and Modeling of Coir Isolator

Figure 6 demonstrates the challenges with processing the coir isolator data. The overall strategy to fit into the proposed model assembly in Figure 2 is outlined in Section 4.1. The need and remedy to recover the large amount of unmeasured cycles are presented in Section 4.2. The identification procedure is given in Section 4.3. Eventually, the identified model assembly is given in Section 4.4.

### 4.1. Analysis

**Figure 11:**
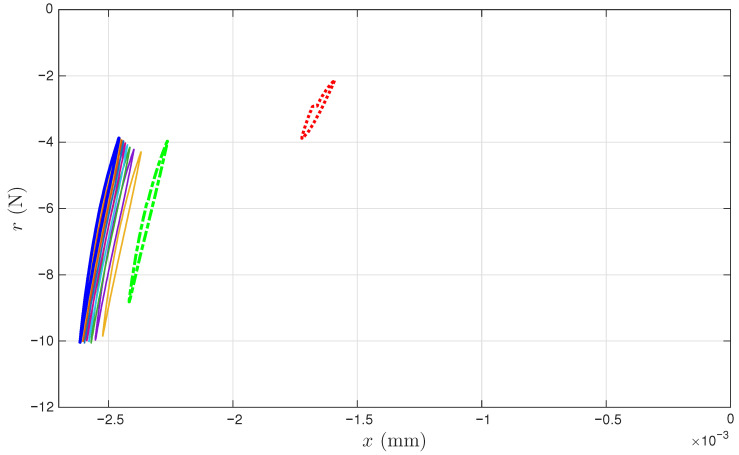
For the coir isolator: The raw data considering the entire loading history with the origin included. The measured cycles are 1 (red), 102 (green), 204, 300, 402, 501, 600, 702, 801, 900, and 1000 (blue).

**Figure 12:**
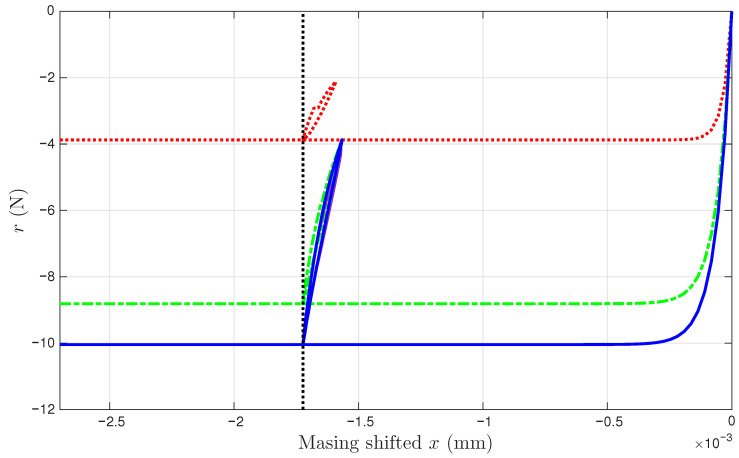
For the coir isolator: The measured first cycle (red) and proposed intermediate responses for the cycles 102 (green), 204, 300, 402, 501, 600, 702, 801, 900 and 1000 (blue) of the underlying Masing model that evolves with time.

**Figure 13:**
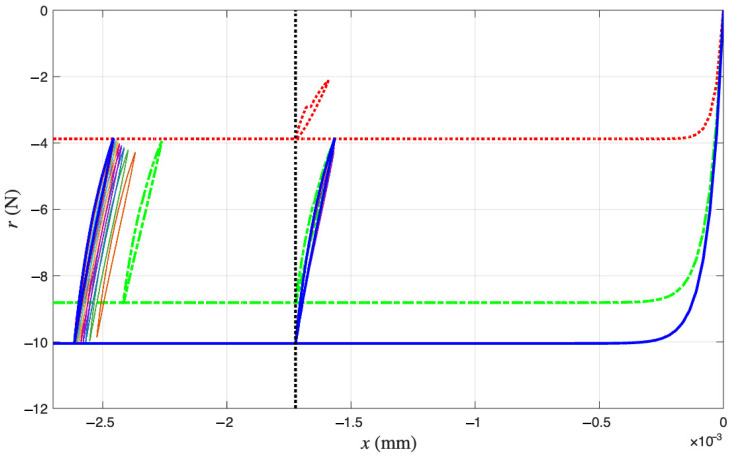
For the coir isolator: The measured first cycle (red) and proposed intermediate responses for the cycles of 102 (green), 204, 300, 402, 501, 600, 702, 801, 900 and 1000 (blue) of the underlying Masing model that evolves with time as in Figure 12, and the further ratcheted cycles of 102, 204, 300, 402, 501, 600, 702, 801, 900 and 1000 to recover their measured counterparts as in Figure 11.

All missing loops are ignored for the time being so that we can focus on the overall picture. The trio of Figure 11, Figure 12 and Figure 13 is designed to illuminate the modeling idea using the model assembly in Figure 2.

Figure 11 presents the measured hysteresis loops in compression only. We do not know how we got there, as the process was not being completely recorded, but we assume that the experiment starts with zero displacement and zero force. Highlighted in thickened lines are the first, second, and last recorded loops in red, green, and blue, which are the 1st, 102nd, and 1000th cycle, respectively. These cycles are minor loops of the Masing model that we are building.

The first recorded loop in red is the first minor loop of the time-varying Masing model at that time. Since the minor loop is measured, we can reconstruct the corresponding virgin loading curve based on the Masing Rule 1 as reviewed in Section 1.3 and by following the four steps below. The reconstructed virgin loading curve is colored in red in Figure 12.

1. The virgin loading curve goes through zero displacement and zero force.
2. Referring to Figure 6a,c, the minimum displacement point (to be approximated with the 6001st point later) in a segmented measurement is where the minor loop closes on the virgin loading curve because it is a velocity turning point.
3. The initial tangent stiffness of the unloading branch of the minor loop is equal to *K*, the initial tangent stiffness of the virgin loading curve.
4. We assume that the yield plateau is equal to the *r* value at the minimum displacement point (to be approximated with the 6001st point later). That is, the Masing model is yielded when the minor loop starts to take place. This is one of the assumptions made to overcome the unidentifiable difficulty stated in Section 1.3. This is the major assumption made for $g_{2}\left(a_{M}\right)$ mentioned at the end of Section 3.2.

If the coir isolator’s cyclic response followed Equation (1), i.e., being the original time-invariant extended Masing model from [20], then the cyclic loading with quite well-controlled displacement amplitude as shown in Figure 6 would lead to repetitive minor loops just like the first minor loop. That is, the remaining minor loops would stay at the position of the first minor loop, colored in red. This prediction is in stark contrast to the measured loops, hinting at the inadequacy of the extended Masing model.

The second recorded loop in green is the 102nd minor loop of the time-varying Masing model at that time. Since the minor loop is measured, we can reconstruct the corresponding virgin loading curve by repeating the above four steps. The reconstructed virgin loading curve is colored in green in Figure 12.

We continue like this until reaching the last recorded loop in blue, which is the 1000th minor loop of the time-varying Masing model at that time. Since the minor loop is measured, we can reconstruct the corresponding virgin loading curve by repeating the above four steps. The reconstructed virgin loading curve is colored in blue in Figure 12.

This explains Figure 12, which justifies why a time-varying Masing model is needed for the coir isolator’s cyclic response, one of the modeling components in Figure 2.

Contrasting Figure 12 with Figure 11, we have Figure 13, which shows that the second recorded loop in green drifts to the left horizontally, i.e., having more compressive deformation. Other recorded loops keep drifting to the left horizontally until the last recorded loop in blue is drifting the most. These drifts are displacement ratcheting to be modeled by a mem-dashpot, the other modeling component in Figure 2. We assume that the displacement ratcheting starts at the closure of the first minor loop. This assumption may not be accurate; however, it is another assumption made to overcome the unidentifiable difficulty stated in Section 1.3.

To summarize this overall analysis, there are two simultaneous processes going on.

First, a flow-controlled input leads to minor loops that are drifted and stretched vertically because the Masing model is strengthened from cycle to cycle due to compaction. It can be seen that the Masing model evolves with time, which explains why Equation (31) is devised to capture this time evolution using absement-based $g_{1}\left(a_{M}\right)$ and $g_{2}\left(a_{M}\right)$, the two nonlinear functions to be identified for this flow-controlled setting.

Next, within every minor loop, displacement ratcheting comes into play, showing up in the horizontal drift of the minor loops. This explains where to apply Equation (4). The displacement ratcheting is a nonlinear function of the generalized momentum as introduced in Equations (13) and (19).

In summary, the trio of Figure 11, Figure 12 and Figure 13 is designed to show how all 1000 minor loops of the extended Masing model, which are of the same shape and share the same closure point, ($x^{*}$, $r^{*}$), have become 1000 individual loops with the same width but increasing height, and at different latitude and longitude.

### 4.2. Approximating Unmeasured Cycles

**Figure 14:**
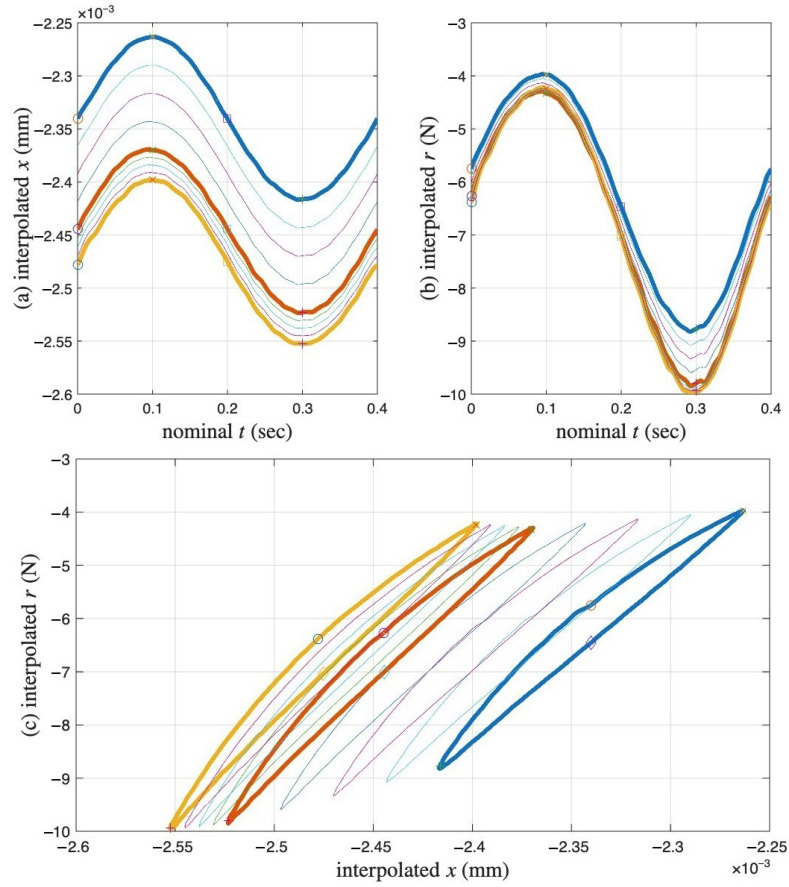
For the coir isolator: A numerical example to illustrate and validate the linear interpolation code in this study to approximate the missing cycles. Three cycles each, using thin lines, are added uniformly between the measured second and third, and the third and fourth cycles, which are in thick lines. (**a**) Segmented *x* time histories, (**b**) segmented *r* time histories, and (**c**) the corresponding hysteresis loops, where the darkened segmented time histories and hysteresis loops are measured.

Not all cycles are measured, which prompts the need of approximating the missing cycles so that the integrated time histories can be obtained to use mem-models. For the measured cycles, the sampling rate is 20 kHz. For a period of 0.4 s, there are 8001 points per recorded cycle, or segmented time history. Each cycle repeats the same time, which is nominal. The actual continuous time vector can be recovered only after all missing cycles are recovered and in the correct order. Linear interpolation is carried out to approximate those missing cycles, which are named interpolated segmented time histories. This means that for every point of the 8001 points, we insert uniformly those missing points. See Figure 14 to explain and validate the adopted linear interpolation using a hypocritically assumed (very small) number of missing cycles so that the added loops can be visualized.

**Figure 15:**
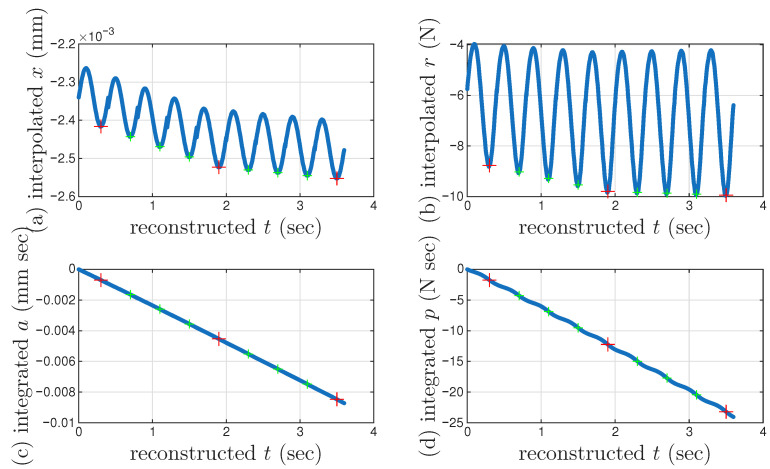
For the coir isolator: The numerical example to illustrate and validate the linear interpolation code in this study to approximate the missing cycles to continue with that in Figure 14. Three cycles each are added uniformly between the measured second and third, and the third and fourth cycles. The interpolated segmented time histories shown in Figure 14 are stitched together to form the approximated (**a**) x(t), and (**b**) r(t), which are further numerically integrated for (**c**) a(t), and (**d**) p(t), respectively. Crosses mark the 6001st point of every cycle, which is an approximation of the velocity turning point for minor loop closure. The red and green are for a measured and interpolated cycle, respectively.

We continue with Figure 15 to illustrate how to recover the full time histories of displacement and restoring force. Once all missing cycles are interpolated to both *x* versus nominal *t* and *r* versus nominal *t* as in Figure 14 to form the interpolated segmented time histories, they are stitched together to form reconstructed *t* and the corresponding *x* and *r* so that the full time histories of x(t) and r(t) are approximated. Figure 15a,b present these approximated time histories, which do not appear continuous due to the three cycles added for illustration purpose. The actual numbers of the missing cycles are 102 and 96 between the measured second and third, and the third and fourth cycles, respectively.

It is of paramount importance to identify the velocity turning point for minor loop closure for every cycle. For the measured 11 cycles, the monotonicity is confirmed so that the minimum displacement point is used as the velocity turning point as reported in Section 2.1. For the interpolated cycles, the accuracy of using the minimum displacement point is questionable due to the error caused by the adopted linear interpolation. Given this concern and for overall efficiency, the 6001st point of every cycle (measured and interpolated) is used as an approximation of the velocity turning point for minor loop closure.

The corresponding time integrals of x(t) and r(t) are produced subsequently, see the integrated a(t) and p(t) in Figure 15c,d, respectively. It can be seen that *a* and *p* are monotonic functions of time. This is why we use *a* and *p* for time parameterization.

The first limitation with this example is that it starts with the measured second cycle, which is the 102nd minor loop. The reconstructed *t* should not start with zero. The time integrals in Figure 15c,d both use zero initial value, which should be some negative values from the minor loops before. The next limitation with this example is that it does not add all missing cycles so that the duration of the reconstructed *t* is much shorter than the actual situation. The ranges for both the time integrals in Figure 15c,d are much shorter than the actual situation too.

### 4.3. Identification Procedure

Bearing these limitations of Figure 15 in mind and using the trio of Figure 11, Figure 12 and Figure 13, we can think about the flow-controlled coir test with an intention to iterate 1000 minor loops at the same minor loop closure point, ($x^{*}$, $r^{*}$), with the same amplitude of displacement, after traveling through the virgin loading curve that is not measured. The reality is more complicated: First, because the Masing model is hardened with increased $r_{u}$ and *K* cycle after cycle, the latitude of the minor loop closure point shifts and the required amplitude of restoring force increases from cycle to cycle. Next, because the dashpot starts to contribute after the closure of the first minor loop as assumed, the longitude of the minor loops shifts cycle after cycle. This is a recap of the analysis of the trio of Figure 11, Figure 12 and Figure 13.

All these increments and shifts are smooth functions of time. The time integrals of displacement and restoring force, called absement and generalized momentum, respectively, are monotonic functions as demonstrated in Figure 15c,d. We will thus quantify these increments and shifts as functions of absement or generalized momentum, which is the identification.

The overall identification procedure is to reverse the analysis procedure of the trio of Figure 11, Figure 12 and Figure 13:

- Collect all minor loop closure points, the 6001st point of every cycle (including both segmented measurements and interpolated), marked with a cross +. Since the Masing minor loops close at these points, we only observe the displacement ratcheting. Because we assume that the displacement ratcheting starts at the closure point of the first minor loop, the coordinates of the closure point of the first minor loop are $x^{*}$ and $r^{*}$. The *x* values of these 1000 points will be minus the *x* value of the first point throughout to obtain the $x_{d}$ values of these 1000 points. The *p* values of these 1000 points are ready. The nonlinear one-to-one mapping of $F_{d}\left(p\right)$ as in Equation (19) will be ready to be identified by curve fitting.
- Use the approximated full time history of p(t) to interpolate the pair of *p* and $x_{d}$ of the 1000 points to obtain a full time history of $x_{d}\left(t\right)$.
- Use the approximated full time histories of *x* and $x_{d}$ to compute a full time history of $x_{M}=x-x_{d}$, which is called the approximated full time history of $x_{M}$.
- Integrate the approximated full time history of $x_{M}\left(t\right)$ for $a_{M}\left(t\right)$, which is called the approximated full time history of $a_{M}$.
- Estimate $g_{1}\left(a_{M}\right)$ using the 6001st point of every cycle (including both segmented measurements and interpolated). Because we assume that the yield plateau is equal to the *r* value at such a point, collect all these 1000 *r* values. The $a_{M}$ values of these 1000 points are ready. The nonlinear one-to-one mapping of $g_{1}\left(a_{M}\right)$ will be ready to be identified by curve fitting.
- Estimate $g_{2}\left(a_{M}\right)$ using the 6001st point of every cycle (including both segmented measurements and interpolated). Because when $r_{M}\to r^{*}$, we have $\frac{dr_{M}}{dx_{M}}\to K$ based on Equation (33), compute all these 1000 $\frac{dr}{dx_{M}}$ values when $r\to r^{*}$, or equivalently, $x_{M}\to x^{*}$. The $a_{M}$ values of these 1000 points are ready. The nonlinear one-to-one mapping of $g_{2}\left(a_{M}\right)$ will be ready to be identified by curve fitting.

### 4.4. Results

**Figure 16:**
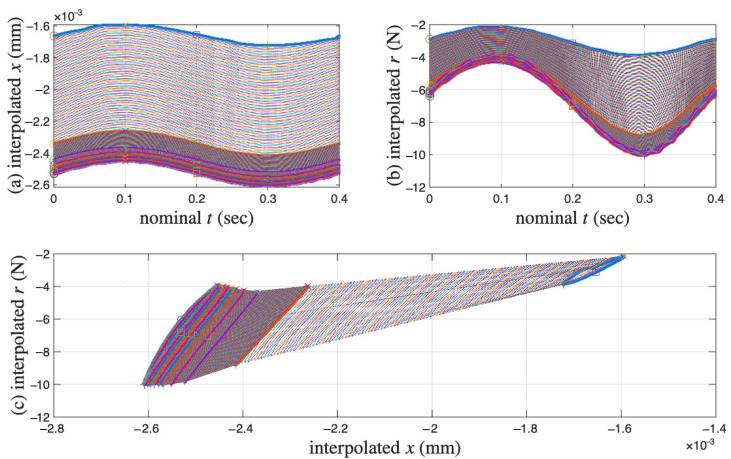
For the coir isolator: The measured and linearly interpolated segmented time histories of (**a**) x(t), and (**b**) r(t), and (**c**) the reconstructed hysteresis loops. The darkened cycles are the measured 11 cycles.

**Figure 17:**
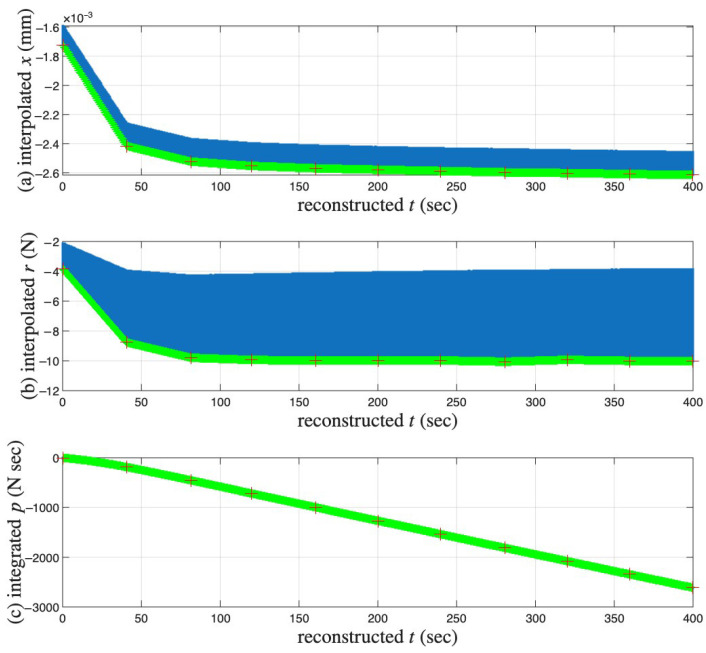
For the coir isolator: The interpolated segmented time histories shown in Figure 16 are stitched together to form the approximated (**a**) x(t), and (**b**) r(t). r(t) is further numerically integrated for (**c**) p(t). Crosses mark the 6001st point of every cycle, which is an approximation of the velocity turning point for minor loop closure. The red and green are for a measured and interpolated cycle, respectively.

The measured and linearly interpolated segmented time histories of x(t) and r(t) and their corresponding hysteresis loops are presented in Figure 16a–c, respectively. The approximated full time histories of x(t), r(t), and the integrated p(t) are presented in Figure 17a–c, respectively.

**Figure 18:**
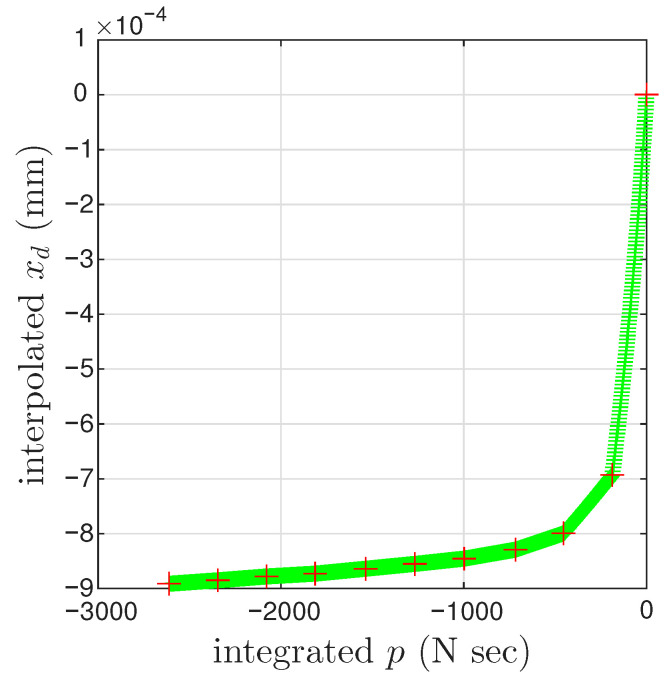
For the coir isolator: The approximated one-to-one mapping $F_{d}\left(p\right)$. Crosses mark the 6001st point of every cycle, which is an approximation of the velocity turning point for minor loop closure. The red and green are for a measured and interpolated cycle, respectively.

Figure 18 presents the results of the identification Step I. The coordinates of the closure point of the first minor loop are $x_{d}=0$ and p<0, which may be hard to see. The nonzero *p* value is resulted from the time integral of *r* from the first to the 6001st point of the measured first cycle. This *p* value is not accurate due to the unmeasured virgin loading curve.

The identification Step II is carried out differently considering the error caused by the adopted linear interpolation. As a matter of fact, the unmeasured loops happens for a reason: the cycle-to-cycle drift is small compared with the displacement amplitude of each cycle. This means that, in $x=x_{M}+x_{d}$ within a cycle, $x_{M}\gg x_{d}$, which can be seen in Figure 16c. With this realization, $x_{d}$ in each cycle follows the $x_{d}$ value of the 6001st point of that cycle.

**Figure 19:**
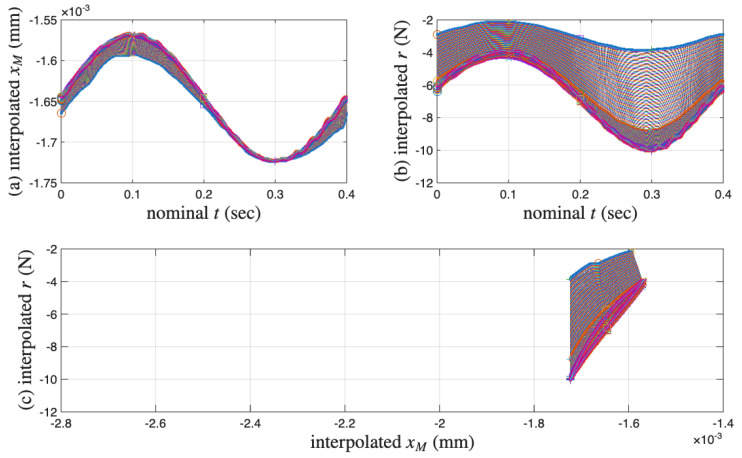
For the coir isolator: The measured and linearly interpolated segmented time histories of (**a**) $x_{M}$, (**b**) *r*, and (**c**) the reconstructed hysteresis loops for the Masing model component of the model. The darkened cycles are the measured 11 cycles.

**Figure 20:**
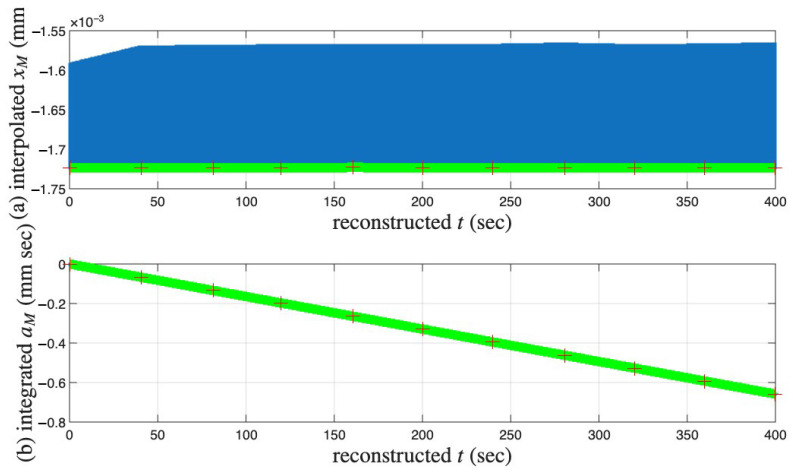
For the coir isolator: The interpolated segmented time histories shown in Figure 19a are stitched together to form the approximated (**a**) $x_{M}\left(t\right)$, which is further numerically integrated for (**b**) $a_{M}\left(t\right)$. The interpolated segmented time histories shown in Figure 19b are stitched together to form the approximated r(t), which is further numerically integrated for p(t), which are the same as those in Figure 17b,c, respectively. Crosses mark the 6001st point of every cycle, which is an approximation of the velocity turning point for minor loop closure. The red and green are for a measured and interpolated cycle, respectively.

Figure 19 and Figure 20 present the results of the identification Steps III and IV, respectively.

**Figure 21:**
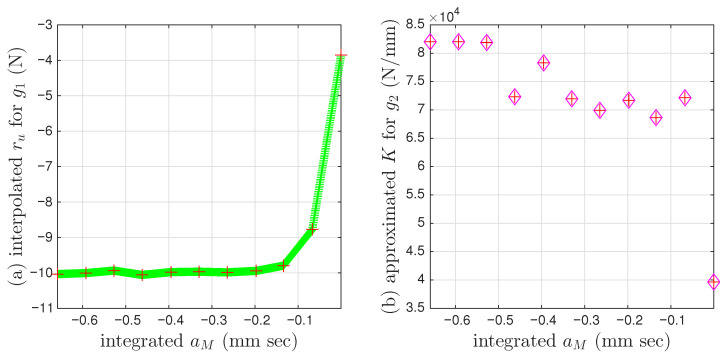
For the coir isolator: (**a**) The approximated $g_{1}\left(a_{M}\right)$. Crosses mark the 6001st point of every cycle, which is an approximation of the velocity turning point for minor loop closure. The red and green are for a measured and interpolated cycle, respectively, and (**b**) the approximated $g_{2}\left(a_{M}\right)$. The 11 points have the $a_{M}$ values of the 6001st point of each measured cycle and the maximum $\frac{dr}{dx_{M}}$ value of the same cycle from Figure 22 with a 200-point moving average.

**Figure 22:**
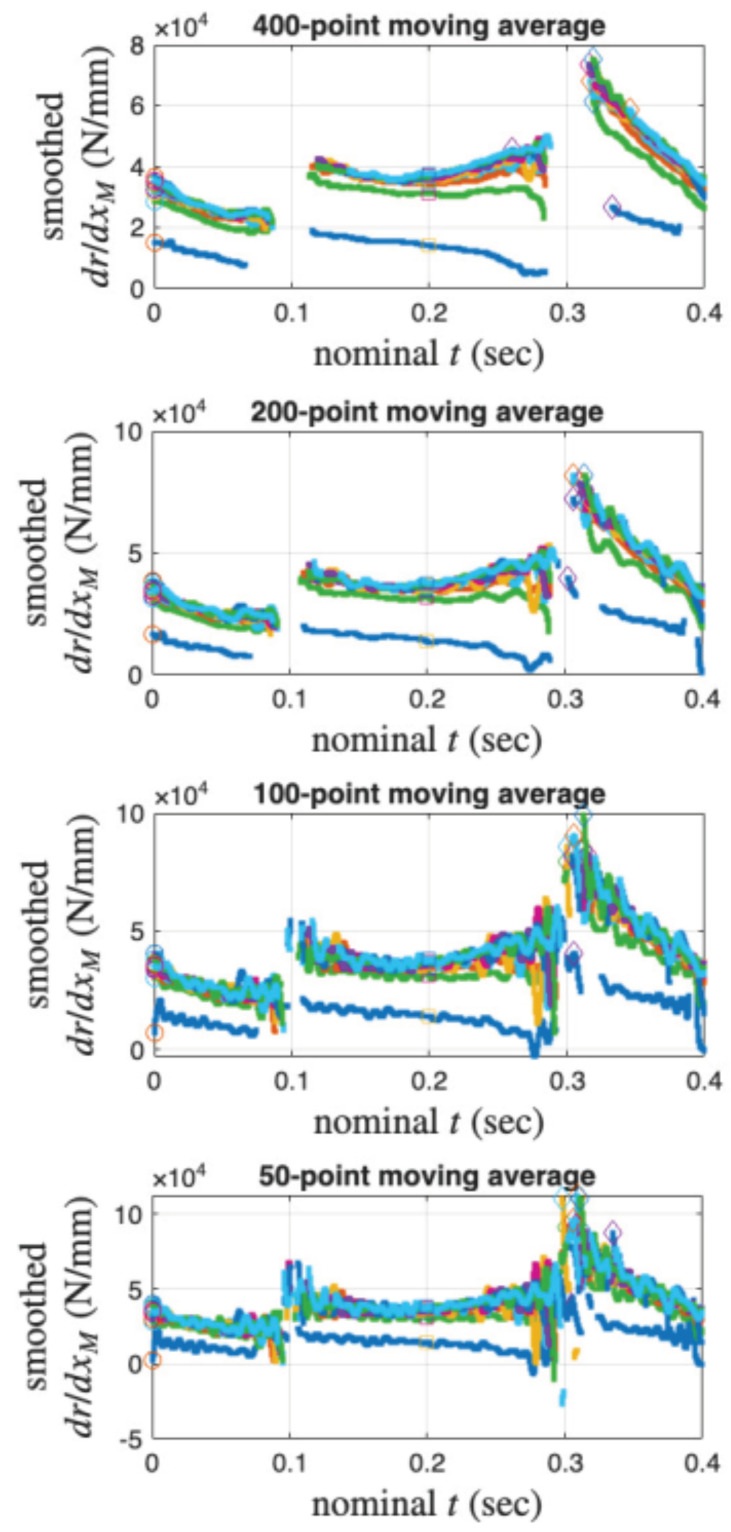
For the coir isolator: A parametric study to smooth $\frac{dr}{dx_{M}}$ using moving average using the measured 11 cycles.

The identification Step V is straightforward; the result is presented in Figure 21a. The identification Step VI has a practical limitation so that it is carried out differently. The result is presented in Figure 21b, and the details are given in Figure 22 and explained as follows:

Recall that the 6001st point of every cycle (measured and interpolated) is used as an approximation of the velocity turning point for minor loop closure. Unfortunately, the 6001st point does not appear to be a good choice to approximate $\frac{dr}{dx_{M}}$ values when $r\to r^{*}$. Numerically, $\frac{dr}{dx_{M}}$ is computed as $\dot{r}$ divided by ${\dot{x}}_{M}$. Figure 22 presents the time histories of $\frac{dr}{dx_{M}}$ of the measured 11 cycles with an intention to locate the maximum $\frac{dr}{dx_{M}}$ value per cycle. The moving average is used to smooth the time histories. Figure 22 indicates that the choice of 200-point moving average may be the most proper. Figure 21b uses the $a_{M}$ value of the 6001st point of every measured cycle and the maximum $\frac{dr}{dx_{M}}$ value of the same cycle (but not necessary at the 6001st point).

The non-smooth nature of the curves in Figure 17, Figure 18, Figure 20 and Figure 21 is because only a small selection of the cycles undergone by the specimen were recorded. In this work, linear interpolation was used between measurement points, which therefore appear as straight lines between the actual measurement points.

## 5. Modeling of Soil Specimens

**Figure 23:**
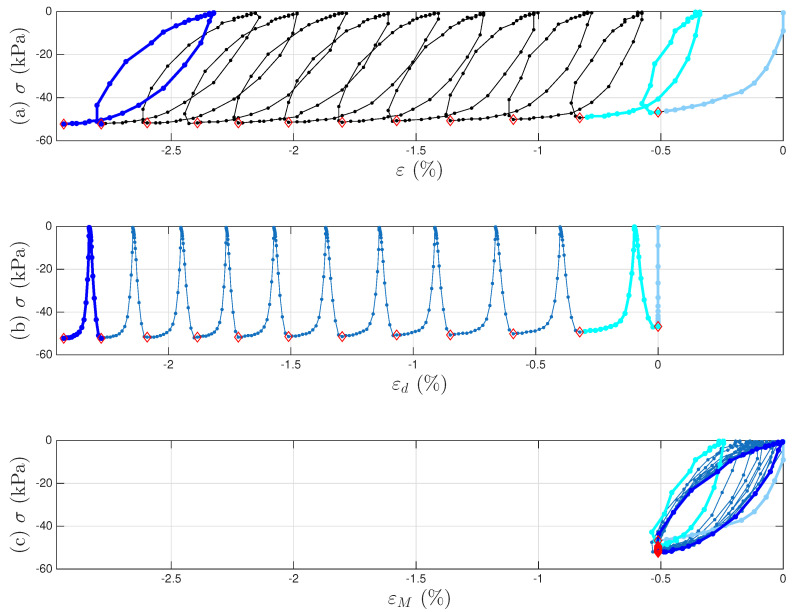
For Test 3 of the soil data: the strain decomposition scheme leading to Figure 1, Figure 7, Figure 8, Figure 9 and Figure 10 and Figure 24 using the first 11 cycles for illustration. (**a**) The measured hysteresis loops, and the corresponding hysteresis loops to be modeled by (**b**) the mem-dashpot for the inter-cycle, and (**c**) the enriched Masing model for the intra-cycle responses resulted from the strain decomposition. The virgin loading curve, and the 1st and 11th intra-cycles are colored in baby blue, cyan, and blue, respectively. The identified minor loop closure points are marked with red diamonds.

To illustrate the strain decomposition details leading to Figure 1 and Figure 7, Figure 8, Figure 9 and Figure 10. Figure 23a presents the measured first 11 cycles of Test 3 of the soil data. All minor loop closure points need to be identified, which follows the algorithm introduced in [9]. The local peaks of $\dot{\sigma }\left(t\right)$ are found first, and an empirically determined offset value for all these time instants is then applied to finalize the time instants for minor loop closure. The same offset values for the two tests are used as in [9]. These identified minor loop closure points are marked with red diamonds in Figure 23.

**Figure 24:**
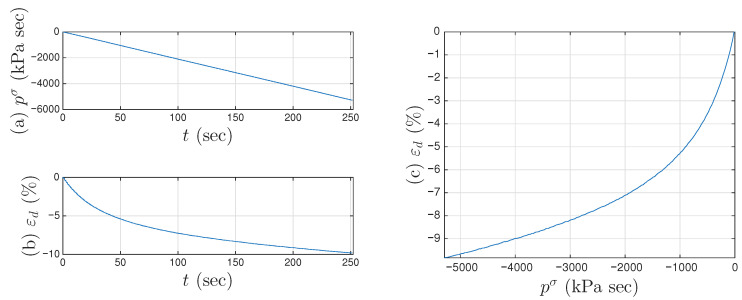
For Test 3 of the soil data: (**a**) the integrated full time history of $p^{\sigma }$, (**b**) the approximated full time history of $x_{d}$, and (**c**) the approximated one-to-one mapping $F_{d}\left(p^{\sigma }\right)$.

We continue to assume that the displacement ratcheting starts at the closure of the first minor loop as shown in Figure 23b. Step I of the procedure in Section 4.3 leads to the identified mem-dashpot. Step II of the procedure in Section 4.3 is further followed, leading to a full time history of $\varepsilon_{d}\left(t\right)$. Figure 24a,b present the full time histories of $p^{\sigma }\left(t\right)$ and $\varepsilon_{d}\left(t\right)$, respectively. Figure 24c is the approximated one-to-one mapping $F_{d}\left(p^{\sigma }\right)$ for Test 3 of the soil data, the counterpart of the approximated one-to-one-mapping $F_{d}\left(p\right)$ for the coir isolator in Figure 18. Steps III and IV of the procedure in Section 4.3 are followed next, leading to the full time histories of $\varepsilon_{M}\left(t\right)$ and $a_{M}^{\varepsilon }\left(t\right)$. With the identified minor loop closure points, Steps V and VI of the procedure in Section 4.3 are further followed, using either $a_{M}^{\varepsilon }\left(t\right)$ or $p_{M}^{\sigma }\left(t\right)$ (which is equal to $p^{\sigma }\left(t\right)$ based on Equation (29)). Figure 10a,b are the approximated $f_{1}\left(p_{M}^{\sigma }\right)$ and $f_{2}\left(p_{M}^{\sigma }\right)$, respectively for Test 3 of the soil data. Their counterparts of the approximated $g_{1}\left(a_{M}\right)$ and $g_{2}\left(a_{M}\right)$ for the coir isolator are in Figure 21a,b, respectively.

**Figure 25:**
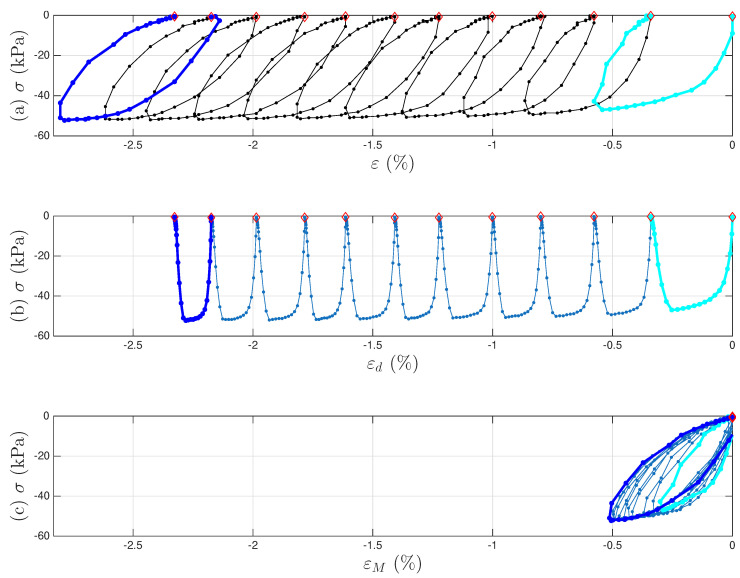
For Test 3 of the soil data: An alternative strain decomposition scheme leading to the results in [9] using the first 11 cycles for illustration. (**a**) The measured hysteresis loops, and the corresponding hysteresis loops to be modeled by (**b**) the mem-dashpot for the inter-cycle, and (**c**) the enriched Masing model for the intra-cycle responses resulted from the strain decomposition. The 1st and 11th intra-cycles are colored in cyan and blue, respectively. The identified minor loop closure points are marked with red diamonds.

The Masing Rule 1 in [12] does not apply the soil datasets well. An alternative treatment is to use the minor loops of a Masing model only. The strain decomposition to fulfill this treatment is illustrated in Figure 25, in contrast to Figure 23. Using the positive sign for compression, the resulted one-to-one mapping $F_{d}\left(p^{\sigma }\right)$, approximated as $f_{1}\left(p_{M}^{\sigma }\right)$ and $f_{2}\left(p_{M}^{\sigma }\right)$ of this alternative modeling treatment is reported in [9]. Ref. [9] continues to plug these identified characteristics back into the model assembly as in Figure 2 for a full-system dynamic simulation.

## 6. Discussions

The results considered in Section 4 and Section 5 show that the modeling approach works effectively in representing the behavior of two material types which are physically very different but which both display micro-interface slip damping. The enriched Masing model captures changes to the hysteresis loop, while the mem-dashpot accounts for the drift between cycles. This section considers the significance of the findings and improvements that we intend to make in the future.

The data from the compressed coir fiber testing provided challenges, in that measurements were only recorded for various distinct cycles. Testing was carried out using equipment that provided amplitude control rather than waveform control, and the virgin loading curve was not available. The linear interpolation between measurement times was acceptable because the properties of the specimen changed only gradually—there were no sudden changes in behavior. In future work, the testing will be conducted using full waveform control and measurements will be retained for consecutive cycles to avoid the need for interpolation when fitting the enriched Masing model. Following the identification procedure listed under Section 4.3, we will have a more accurately identified minor loop closure points using the measured consecutive loops for the identification Step I. We will then be able to reconstruct a complete time history of $x_{d}\left(t\right)$ for those measured consecutive loops as in Step II. The approximation accuracy for $g_{2}\left(a_{M}\right)$ as in Figure 21b will be improved under Step VI. We will then be able to examine whether the asymmetric individual loops can be better explained and modeled using the proposed model assembly as in Figure 2.

In [12], the generalized Masing has been investigated under various amplitude–modulate sine waves (in Figure 3 there) and an El Centro ground motion time history (in Figure 24 there). We thus anticipate the same capability of the proposed enriched generalized Masing model. However, specific test data undertaken using complex loading history would be needed to validate this. This requires a new experimental campaign—something we intend to do in the future.

The proposed models describe behavior using a small number of fitting parameters that capture the basic physics of the processes involved. As a result, it should be possible to identify the sensitivity of these parameters to manufacturing and test conditions. As the compressed coir fiber isolator has not been reported in the literature before, understanding the sensitivity to important parameters will be critical for designers to be able to work with this medium. Important factors relating to manufacture include fiber variability; manufacturing conditions including temperature, pressure, moisture and lay-up; and environmental conditions such as the water content of the fibers and presence of contaminants. Similarly, test conditions for further investigation include the loading regime (shear or compression); the vibration time history including cyclic frequency and amplitude; and any steady or long-term load. Understanding sensitivity to such factors will allow the model to incorporate statistic variability, making the prediction of behavior more reliable.

## 7. Summary

This paper has shown how to model a damper employing micro-interface friction where properties change according to usage. The device studied is a novel vibration isolator constructed from coconut fibers (coir). We seek simple and physically insightful models facing the complex behaviors to be modeled. The solution strategy is to tap from mem-models an emerging family of hysteresis models to enrich the extended Masing model. Such a model has been applied to two different micro-interface damping systems, one coconut fiber isolator and two soil samples. Future work has been identified.

## Figures and Tables

**Figure A1:**
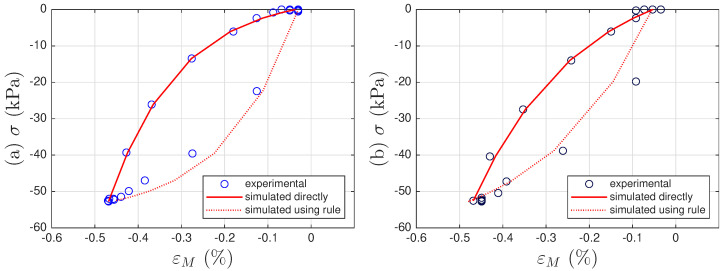
For Test 2 of the soil data: (**a**) The 11th, and (**b**) 200th intra-cycle hysteresis loops fitted using the unloading branch in Equation (40) to be presented later.

**Figure A2:**
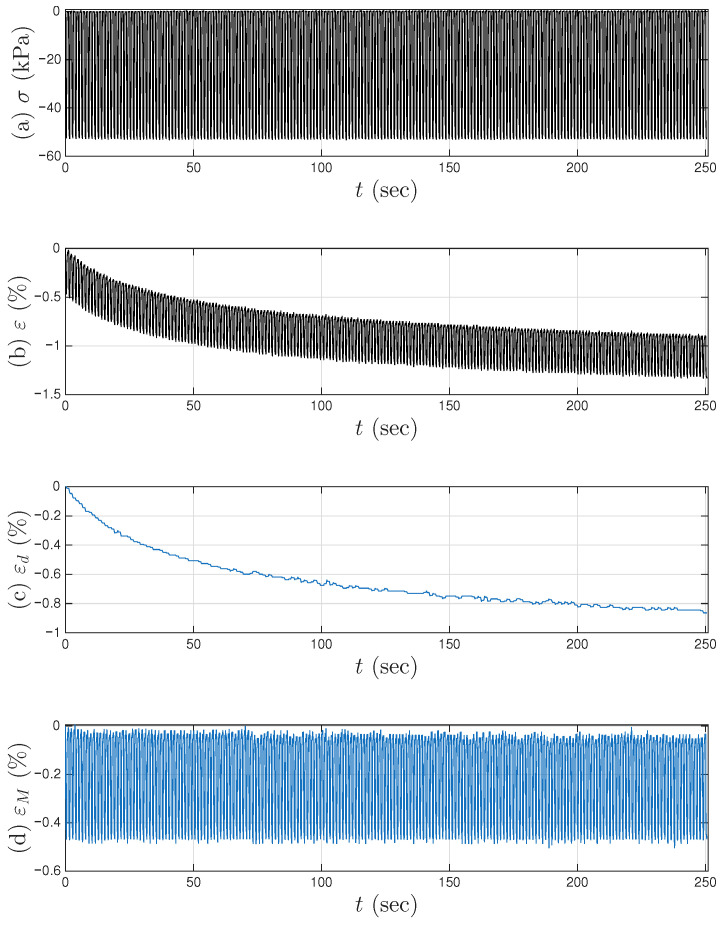
For Test 2 of the soil data: (**a**) the measured stress time history; (**b**) the measured strain time history; and the decomposed strain time history for (**c**) the mem-dashpot for the inter-cycle, and (**d**) the enriched Masing model for the intra-cycle responses.

**Figure A3:**
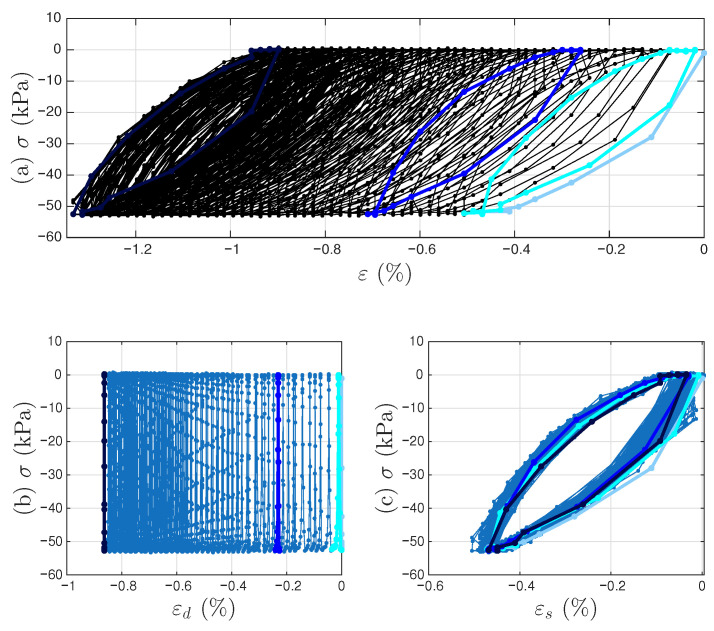
For Test 2 of the soil data: (**a**) the measured hysteresis loops of up to the 200th cycles, and the corresponding hysteresis loops to be modeled by (**b**) the mem-dashpot for the inter-cycle, and (**c**) the enriched Masing model for the intra-cycle responses resulted from decomposing the measured strain time history. The 1st, 11th, and 200th cycles are highlighted in cyan, blue, and dark blue, respectively.

**Figure A4:**
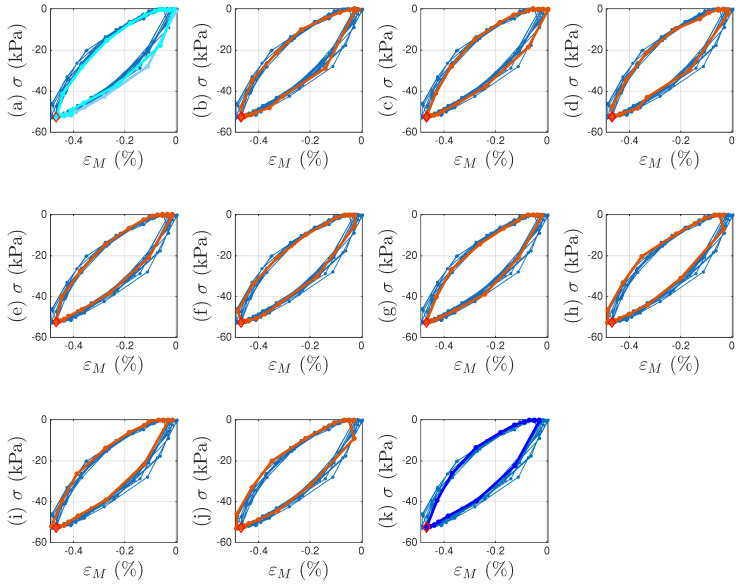
For Test 2 of the soil data: The first 11 intra-cycle hysteresis loops with one loop highlighted at a time following (**a**–**k**) to reveal the virgin loading curve and minor loop closures using Masing model concepts. The virgin loading curve, 1st and 11th intra-cycles, and other intra-cycles in-between are colored in baby blue, cyan, blue, and red, respectively.

**Figure A5:**
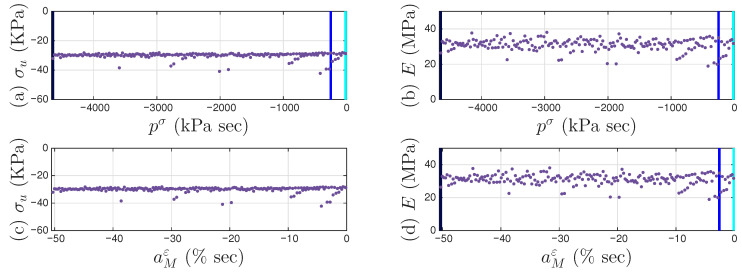
For Test 2 of the soil data: The two parameters, yield strength $\sigma_{u}$ (**a**,**c**) and initial stiffness *E* (**b**,**d**), as affected by generalized momentum $p^{\sigma }$ (**a**,**b**) and absement $a_{M}^{\varepsilon }$ (**c**,**d**) for the first 200 intra-cycles. These intra-cycles are to be modeled by a mem-spring, the enriched Masing model. The 1st, 11th, and 200th intra-cycles are colored in cyan, blue, and dark blue, respectively.

**Figure A6:**
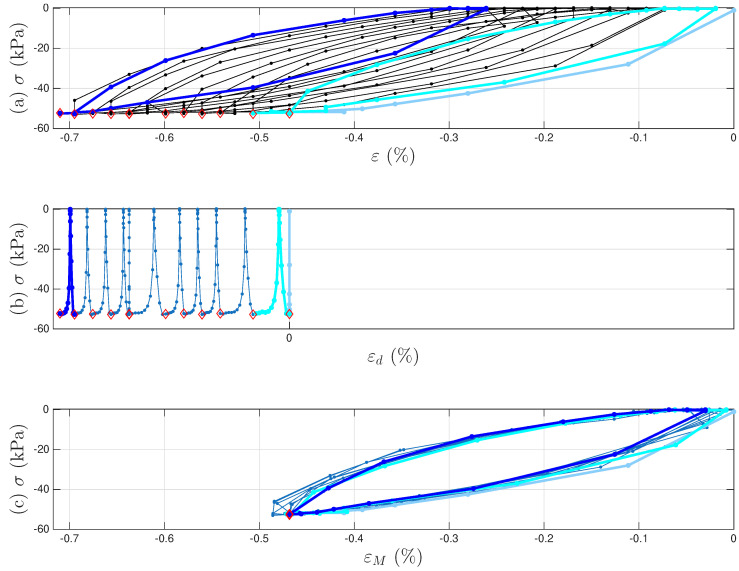
For Test 2 of the soil data: the strain decomposition scheme leading to Figure A1, Figure A2, Figure A3, Figure A4, Figure A5 and Figure A7 using the first 11 cycles for illustration. (**a**) The measured hysteresis loops, and the corresponding hysteresis loops to be modeled by (**b**) the mem-dashpot for the inter-cycle, and (**c**) the enriched Masing model for the intra-cycle responses resulted from the strain decomposition. The virgin loading curve, 1st and 11th intra-cycles are colored in baby blue, cyan, and blue, respectively. The identified minor loop closure points are marked with red diamonds.

**Figure A7:**
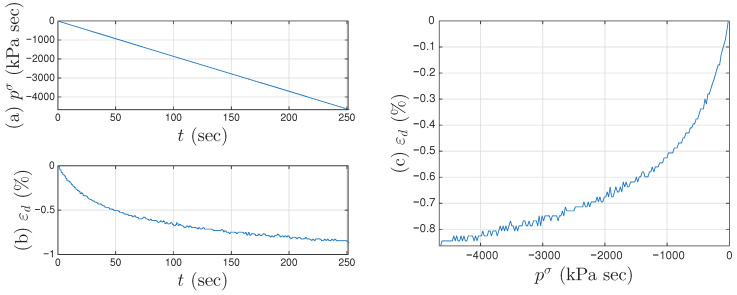
For Test 2 of the soil data: (**a**) the integrated full time history of $p^{\sigma }$, (**b**) the approximated full time history of $x_{d}$, and (**c**) the approximated one-to-one mapping $F_{d}\left(p^{\sigma }\right)$.

**Figure A8:**
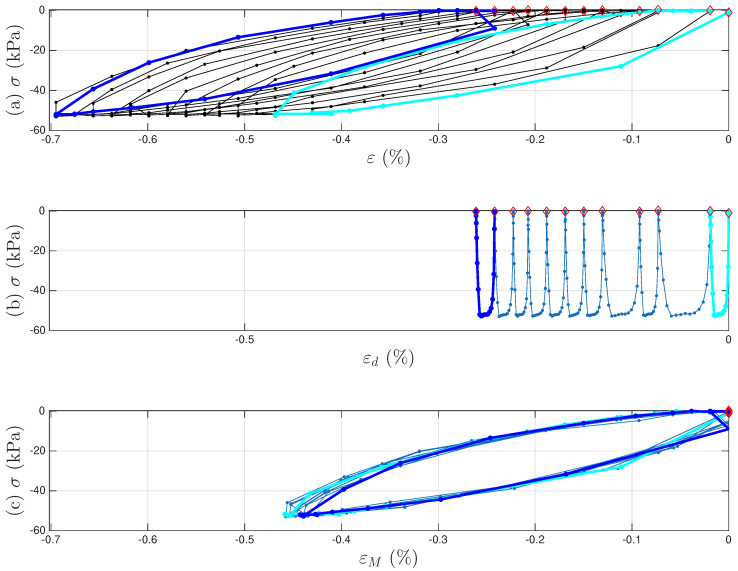
For Test 2 of the soil data: An alternative strain decomposition scheme leading to the results in [9] using the first 11 cycles for illustration. The measured hysteresis loops, and the corresponding hysteresis loops to be modeled by (**b**) the mem-dashpot for the inter-cycle, and (**c**) the enriched Masing model for the intra-cycle responses resulted from the strain decomposition. The 1st and 11th intra-cycles are colored in cyan and blue, respectively. The identified minor loop closure points are marked with red diamonds.

## Data Availability

The original data presented in the study are openly available in OSF at https://osf.io/f7h6p/ (accessed on 21 September 2025) and https://osf.io/phzs9/ (accessed on 21 September 2025) for the coir isolator and soil specimens, respectively. Further requests should be addressed to Jin-Song Pei.

## References

[1] Jones D.I. (2001). Handbook of Viscoelastic Vibration Damping.

[2] Tassini N., Lambrinou K., Mircea I., Bartsch M., Patsias S., Van der Biest O. (2007). Study of the amplitude-dependent mechanical behaviour of yttria-stabilised zirconia thermal barrier coatings. J. Eur. Ceram. Soc..

[3] Chandrasekhar K., Rongong J.A., Cross E. (2019). Mechanical behaviour of tangled metal wire devices. Mech. Syst. Signal Process..

[4] Tinker M.L., Cutchins M.A. (1992). Damping phenomena in a wire rope vibration isolation system. J. Sound Vib..

[5] Panossian H.V. (1992). Structural Damping Enhancement Via Non-Obstructive Particle Damping Technique. J. Vib. Acoust..

[6] Satyanarayana K.G., Kulkarni A.G., Rohatgi P.K. (1981). Structure and properties of coir fibres. Proc. Indian Acad. Sci. Sect. C Eng. Sci..

[7] Perez A., Ferreno D., Carrasco I.A., Polanco J.A., Casado J.A., Diego S. (2020). Metal cushion dampers for railway applications: A review. Constr. Build. Mater..

[8] Miller G., Teh S., Li D., Zaman M. (2000). Cyclic Shear Strength of Soft Railroad Subgrade. ASCE J. Geotech. Geoenviron. Eng..

[9] Pei J.S., Wright J.P., Miller G.A., Gay-Balmaz F., Quadrelli M.B. (2025). Mem-modeling of strain ratcheting using early-time soil fatigue data. Nonlinear Dyn..

[10] Mathis A.T., Balaji N.N., Kuether R.J., Brink A.R., Brake M.R.W., Quinn D.D. (2020). A Review of Damping Models for Structures with Mechanical Joints. Appl. Mech. Rev..

[11] Masing G. Eigenspannungen und Verfestigung beim Messing. Proceedings of the 2nd International Congress for Applied Mechanics.

[12] Beck J.L., Pei J.S. (2022). Demonstrating the power of extended Masing models for hysteresis through model equivalencies and numerical investigation. Nonlinear Dyn..

[13] Caughey T.K. (1960). Random Excitation of a System with Bilinear Hysteresis. J. Appl. Mech..

[14] Caughey T.K. (1960). Sinusoidal Excitation of a System with Bilinear Hysteresis. J. Appl. Mech..

[15] Chiang D.Y., Beck J.L. (1994). A New Class of Distributed-Element Models for Cyclic Plasticity—Part 1: Theory and Application. Int. J. Solids Struct..

[16] Chiang D.Y., Beck J.L. (1994). A New Class of Distributed-Element Models for Cyclic Plasticity—Part 2: On Important Properties of Material Behavior. Int. J. Solids Struct..

[17] Chiang D.Y. (1999). The generalized Masing models for deteriorating hysteresis and cyclic plasticity. Appl. Math. Model..

[18] Ashrafi S.A., Smyth A.W. (2007). A Generalized Masing Approach to Modeling Hysteretic Deteriorating Behavior. ASCE J. Eng. Mech..

[19] Ashrafi S.A., Smyth A.W. (2008). Adaptive Parametric Identification Scheme for a Class of Nondeteriorating and Deteriorating Nonlinear Hysteretic Behavior. ASCE J. Eng. Mech..

[20] Jayakumar P. (1987). Modeling and Identification in Structural Dynamics. Ph.D Thesis.

[21] Jayakumar P., Beck J.L., Natke H.G., Yao J.T.P. (1988). System Identification Using Nonlinear Structural Models. Structural Safety Evaluation Based on System Identification Approaches.

[22] Beck J.L., Jayakumar P. Class of Masing Models for Plastic Hysteresis in Structures. Proceedings of the Proceedings 14th ASCE Structures Congress.

[23] Pei J.S., Gay-Balmaz F., Luscher D.J., Beck J.L., Todd M.D., Wright J.P., Qiao Y., Quadrelli M.B., Farrar C.R., Lieven N.A.J. (2021). Connecting mem-models with classical theories. Nonlinear Dyn..

[24] Ewins D.J. (2000). Modal Testing: Theory, Practice and Application.

[25] Pintelon R., Schoukens J. (2001). System Identification: A Frequency Domain Approach.

[26] Katafygiotis L.S., Beck J.L. (1998). Updating Models and Their Uncertainties. II: Model Identifiability. ASCE J. Eng. Mech..

[27] Pei J.S., Wright J.P., Todd M.D., Masri S.F., Gay-Balmaz F. (2015). Understanding memristors and memcapacitors for engineering mechanical applications. Nonlinear Dyn..

[28] Pei J.S., Gay-Balmaz F., Wright J.P., Todd M.D., Masri S.F. (2017). Dual input-output pairs for modeling hysteresis inspired by mem-models. Nonlinear Dyn..

[29] Chua L.O. (1971). Memrister—The Missing Circuit Element. IEEE Trans. Circuit Theory.

[30] Chua L.O., Kang S.M. (1976). Memristive Devices and Systems. Proc. IEEE.

[31] Strukov D.B., Snider G.S., Stewart D.R., Williams R.S. (2008). The missing memristor found. Nature.

[32] Di Ventra M., Pershin Y.V., Chua L.O. (2009). Circuit Elements with Memory: Memristors, Memcapacitors, and Meminductors. Proc. IEEE.

[33] Oster G.F., Auslander D.M. (1973). The Memristor: A New Bond Graph Element. ASME J. Dyn. Syst. Meas. Control.

[34] Jeltsema D. Memory Elements: A Paradigm Shift in Lagrangian Modeling of Electrical Circuits. Proceedings of the MathMod Conference.

[35] Paynter H.M. (1961). Analysis and Design of Engineering Systems: Class Notes for M.I.T. Course 2.751.

[36] Paynter H.M. (2000). The Gestation and Birth of Bond Graphs. https://sites.utexas.edu/longoria/files/2020/10/Birth_of_-Bond_Graphs.pdf.

[37] Pyke R. (1979). Nonlinear Soil Models for Irregular Cyclic Loadings. J. Geotech. Eng. Div. ASCE.

[38] Pyke R. (2004). Evolution of Soil Models Since the 1970s. International Workshop on Uncertainties in Nonlinear Soil Properties and Their Impact on Modeling Dynamic Soil Response.

[39] The Mathworks, Inc. (2025). MATLAB.

[40] Canfield R. (2015). central_diff.m. http://www.mathworks.com/matlabcentral/fileexchange/12-central-diff-m/content/central_diff.m.

[41] Tran L.Q.N., Minh T.N., Fuentes C.A., Chi T.T., Van Vuure A.W., Verpoest I. (2015). Investigation of microstructure and tensile properties of porous natural coir fibre for use in composite materials. Ind. Crops Prod..

[42] Krasnosel’skiǐ M.A., Pokrovskiǐ A.V. (1983). Systems with Hysteresis.

[43] Lazan B.J. (1968). Damping of Materials and Members in Structural Mechanics.

